# Vascular-derived TGF-β increases in the stem cell niche and perturbs neurogenesis during aging and following irradiation in the adult mouse brain

**DOI:** 10.1002/emmm.201202197

**Published:** 2013-03-25

**Authors:** Jose R Pineda, Mathieu Daynac, Alexandra Chicheportiche, Arantxa Cebrian-Silla, Karine Sii Felice, Jose Manuel Garcia-Verdugo, François D Boussin, Marc-André Mouthon

**Affiliations:** 1 CEA DSV iRCM SCSR, Laboratoire de RadiopathologieFontenay-aux-Roses, France; 2 INSERM, U967Fontenay-aux-Roses, France; 3 Université Paris Diderot, Sorbonne Paris CitéUMR 967, Fontenay-aux-Roses, France; 4 Université Paris SudUMR 967, Fontenay-aux-Roses, France; 5 Laboratorio de Neurobiología Comparada, Instituto Cavanilles, Universidad de ValenciaValencia, CIBERNED, Spain

**Keywords:** aging, endothelial cells, irradiation, neural stem cells, TGF-beta

## Abstract

Neurogenesis decreases during aging and following cranial radiotherapy, causing a progressive cognitive decline that is currently untreatable. However, functional neural stem cells remained present in the subventricular zone of high dose-irradiated and aged mouse brains. We therefore investigated whether alterations in the neurogenic niches are perhaps responsible for the neurogenesis decline. This hypothesis was supported by the absence of proliferation of neural stem cells that were engrafted into the vascular niches of irradiated host brains. Moreover, we observed a marked increase in TGF-β1 production by endothelial cells in the stem cell niche in both middle-aged and irradiated mice. In co-cultures, irradiated brain endothelial cells induced the apoptosis of neural stem/progenitor cells via TGF-β/Smad3 signalling. Strikingly, the blockade of TGF-β signalling *in vivo* using a neutralizing antibody or the selective inhibitor SB-505124 significantly improved neurogenesis in aged and irradiated mice, prevented apoptosis and increased the proliferation of neural stem/progenitor cells. These findings suggest that anti-TGF-β-based therapy may be used for future interventions to prevent neurogenic collapse following radiotherapy or during aging.

## INTRODUCTION

Decreased adult neurogenesis following cranial irradiation, which is a central adjuvant treatment for brain tumours in both paediatric and adult patients, is believed to contribute to cognitive decline (Monje & Palmer, 2003). We and others have reported that exposure of the brain to 15 Gy is accompanied by the perturbation of olfactory memory and is associated with decreased neurogenesis in mice (Lazarini et al, 2009; Valley et al, 2009).

Neural stem cells (NSCs) are located in the adult subventricular zone (SVZ) and are involved in neurogenesis during adulthood (Doetsch et al, 1999). Adult NSCs successively give rise to transit amplifying progenitors (TAPs) and then to neuroblasts, which migrate in chains to the olfactory bulbs (OBs), where they differentiate into neurons (Alvarez-Buylla & Lim, 2004). Dividing NSCs and TAPs establish intimate interactions with blood vessels at sites that lack pericyte coverage to form vascular niches within the adult SVZ (Mirzadeh et al, 2008; Shen et al, 2008; Tavazoie et al, 2008). Increasing evidence has revealed the importance of growth factors that are synthesized by brain endothelial cells (BECs) of the vascular niche in the regulation of neurogenesis, including NSC proliferation (Ramirez-Castillejo et al, 2006). Moreover, molecular cross-talk between NSCs and BECs includes signals that act on both cell types. These signals include members of the vascular endothelial growth factor family (Calvo et al, 2011).

Irradiation provokes apoptosis in proliferating cells in the SVZ and a clear dose-dependent impairment of neurogenesis that is permanent for doses exceeding 10 Gy in rodents (Tada et al, 1999). Whereas some NSCs have been reported to survive after 10 Gy irradiation, they lack the ability to give rise to new neurons (Achanta et al, 2012). Apart from a reduction in the number of resident NSCs, irradiation may also generate a hostile microenvironment. In particular, this treatment may lessen NSC proliferation and differentiation *in vivo*. Indeed, microglial inflammation that accompanies radiation injury has been implicated in neurogenic collapse and NSC dysfunction in the hippocampus (Monje et al, 2003); however, the mechanisms of neurogenesis alteration in the SVZ remain elusive.

Studies indicate that physiological aging is also associated with a progressive reduction in proliferating cells and in doublecortin-positive neuroblasts in the SVZ and OBs of rodents (Enwere et al, 2004; Maslov et al, 2004; Tropepe et al, 1997). A significant decline in neural stem/progenitor cells is apparent by 6 months of age in the SVZ, ultimately resulting in a dramatic reduction in the number of these cells in elderly mice (Enwere et al, 2004). A premature decrease in the NSC pool owing to aging suggests that these NSCs have no self-renewal capacity and/or are programmed to complete only a limited number of divisions (Sii-Felice et al, 2008). However, when the SVZ from aged mice were cultured *in vitro*, NSCs retain their capacity to proliferate and to differentiate into functional neurons, similar to the NSCs in young adult mice, albeit with lower efficiency (Ahlenius et al, 2009; Tropepe et al, 1997). Furthermore, the neurogenesis decline that is observed during ageing in the hippocampus has been attributed in part to changes in the systemic milieu (Villeda et al, 2011).

TGF-β has been widely recognized as an injury-related cytokine, as its levels are strongly and rapidly upregulated in the brain following different forms of injuries (Gomes et al, 2005) and during aging (Werry et al, 2010). The chronic elevation of TGF-β1 triggers accumulation of basement proteins and results in Alzheimer's disease-like cerebrovascular amyloidosis and microvascular degeneration (Wyss-Coray et al, 2000). Although TGF-β promotes the survival of adult neurons (Boche et al, 2003; Schober et al, 2007), it also has an apoptotic effect on proliferating neural-crest-derived multipotent progenitor cells (Hagedorn et al, 2000). Furthermore, TGF-β1 inhibits the proliferation of adult NSCs, although both positive and negative effects of TGF-β1 have been reported on adult neurogenesis (Battista et al, 2006; Buckwalter et al, 2006; Wachs et al, 2006).

This study explores whether the decline in SVZ neurogenesis during aging or following irradiation is merely a function of NSC depletion or reflects more profound changes in the NSC vascular niche. We demonstrate that TGF-β pathway activation was persistently increased in the SVZ niches of irradiated or aged mice. We also report that the selective inhibition of this pathway significantly improved neurogenesis.

## RESULTS

### High-dose radiation decreases neurogenesis but spares NSCs

A mouse model of whole-brain irradiation with a total radiation dose of 15 Gy, divided into three doses of 5 Gy that were delivered at 48 h intervals, was used to explore the effects of radiation on adult neurogenesis in the SVZ. This 15 Gy split-dose irradiation paradigm did not provoke the mobilization or the activation of microglial cells with respect to the number and the resting morphology of CD68^+^ and Iba1^+^ cells in the SVZ/striatum (Supporting Information Fig S1).

As estimated by Ki67-positivity, proliferation was dramatically decreased in the SVZ 4 months following radiation exposure, and the total number of nuclei was also reduced (Supporting Information Fig S2). Despite this reduced proliferation capacity, the survival of NSCs was indicated by the presence of Nestin^+^GFAP^+^ double-positive cells lining the lateral ventricle that were negative for S100β (Supporting Information Fig S3).

**Figure 1:**
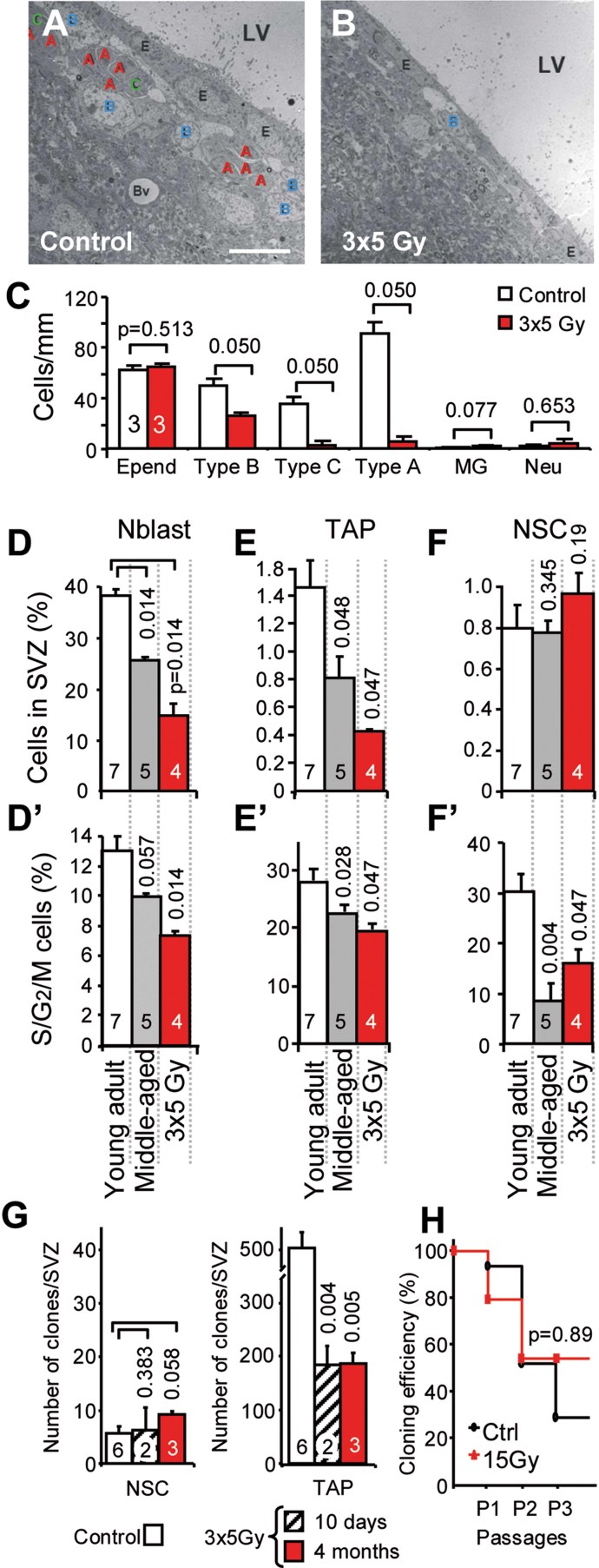
NSCs resisted high radiation exposure but presented proliferation defects. A,B. Electron microscopy revealed the persistence of type B/NSCs 1 year following irradiation in the SVZ, whereas type C/TAPs and type A/neuroblasts were nearly completely lost. Scale bar: 10 µm. C–F. Ependymal cell (Epend), neuron (Neu) and microglial cell (MG) numbers were unaltered. FACS analysis of SVZ populations (percentage in D–F) and their proliferating fraction (DNA >2N in D′–F′): neuroblasts (CD24^+^ in D and D′), TAPs (EGFR^+^ in E and E′) and activated NSCs (LeX^+^EGFR^+^ in F and F′). The *p*-value was determined using the Mann–Whitney *U*-test. G. The quantification of NSCs and TAPs in the N-CFCA. The mean ± SD of two to five independent experiments is shown (the number of mice is indicated within the bars). The *p*-value was determined using the Mann–Whitney *U*-test. H. NSC-derived clones were subcultured to confirm their self-renewal capacity. The Kaplan–Meier's analysis is shown.

We previously reported that this irradiation regimen reduces the number of neuroblasts in the SVZ and decreases their arrival at the OBs, inducing olfactory memory deficits in mice (Lazarini et al, 2009). The drastic decrease in neuroblasts/type A and TAPs/type C cells was also observed 1 year following irradiation (Fig 1B and C). Although their absolute number was decreased, half of the type B cells persisted for 1 year following exposure (Fig 1B and C).

We further examined the content of NSCs and their progeny at 4 months by FACS analysis on freshly dissociated microdissected SVZs. In agreement with the reduction in proliferation described above, the total number of cells in the dissociated SVZ decreased to 25 ± 5 × 10^3^ cells/SVZ 4 months following irradiation compared to 47 ± 7 × 10^3^ cells in young non-irradiated control mice (*p* = 0.019). The total number of SVZ cells also significantly decreased in 12-month-old (*i.e.* middle-aged) mice, reaching 32 ± 4 × 10^3^ cells/SVZ (*p* = 0.032). Given that LeX is expressed in the SVZ on GFAP-positive cells that have NSC features (Capela & Temple, 2002), an anti-LeX antibody was used in combination with CD24 and an EGF fluorescent ligand to label neuroblasts and activated NSCs. According to a previous report (Pastrana et al, 2009), we defined the following three populations: (i) CD24^−^LeX^+^EGFR^+^ activated NSCs, (ii) CD24^−^LeX^−^EGFR^+^ TAPs and (iii) CD24^+^ neuroblasts. The purity of these sorted SVZ populations was confirmed using qRT-PCR for the mRNA expression levels of specific NSC, TAP and neuroblast markers (Supporting Information Fig S4).

As was expected from our previous findings (Lazarini et al, 2009), a decrease in CD24^+^ neuroblasts was observed in irradiated mice (Fig 1D). The percentage of TAPs (EGFR^+^LeX^−^) diminished in the SVZ of both middle-aged and irradiated mice (Fig 1E), whereas the relative number of NSCs (LeX^+^EGFR^+^) was unaltered (Fig 1F). FACS analysis using GLAST, which is another NSC marker that is expressed on nearly all LeX-positive cells (Supporting Information Fig S4D), confirmed that the percentage of CD24^−^GLAST^+^ cells, *i.e.* the population that contained NSCs, was maintained in the SVZ following irradiation and during aging (Supporting Information Fig S5). However, as the total number of cells was reduced in the SVZ, the absolute number of NSCs was decreased following irradiation and during aging; however, this decrease was less pronounced than for their progeny. Moreover, all of these populations exhibited a diminution in their proliferation status (DNA content >2N), being reduced for NSCs by 72% and 47% during aging and following irradiation, respectively (Fig 1D′–F′).

These findings led us to analyse the capacity of SVZ cells from irradiated mice to form neurospheres in the presence of EGF and FGF2 using the neural colony forming cell assay (N-CFCA), which enables NSCs to be discriminated from TAPs based on neurosphere size (Louis et al, 2008). When SVZ cells from irradiated mice were cultured in N-CFCA, small neurospheres that were initiated by TAPs were reduced in number following irradiation, an effect that was observed at both 10 days and 4 months (Fig 1G). In contrast, larger neurospheres that were derived from NSCs were generated with the same efficacy as control mice, suggesting that NSCs resisted this radiation regimen and preserved their capacity to proliferate *in vitro* (Fig 1G). These neurospheres were individually subcultured in neurosphere medium; half of these neurospheres exhibited a capacity for self-renewal for three subsequent passages, with similar efficiencies for the irradiated mice and the controls (Fig 1H).

Therefore, our data demonstrate that radiation induced a dramatic decrease in neurogenesis despite the persistence of functional NSCs, as was previously reported in aged mice (Ahlenius et al, 2009).

### The NSC niche is altered following irradiation

In light of these results, we reasoned that the dramatic decline in neurogenesis may be rooted in an alteration in the NSC microenvironment rather than the intrinsic loss of the NSCs. To test this hypothesis, we used a transplantation model in which neural stem/progenitor cells were grafted into the SVZ of irradiated hosts or control C57Bl6 mice.

**Figure 2:**
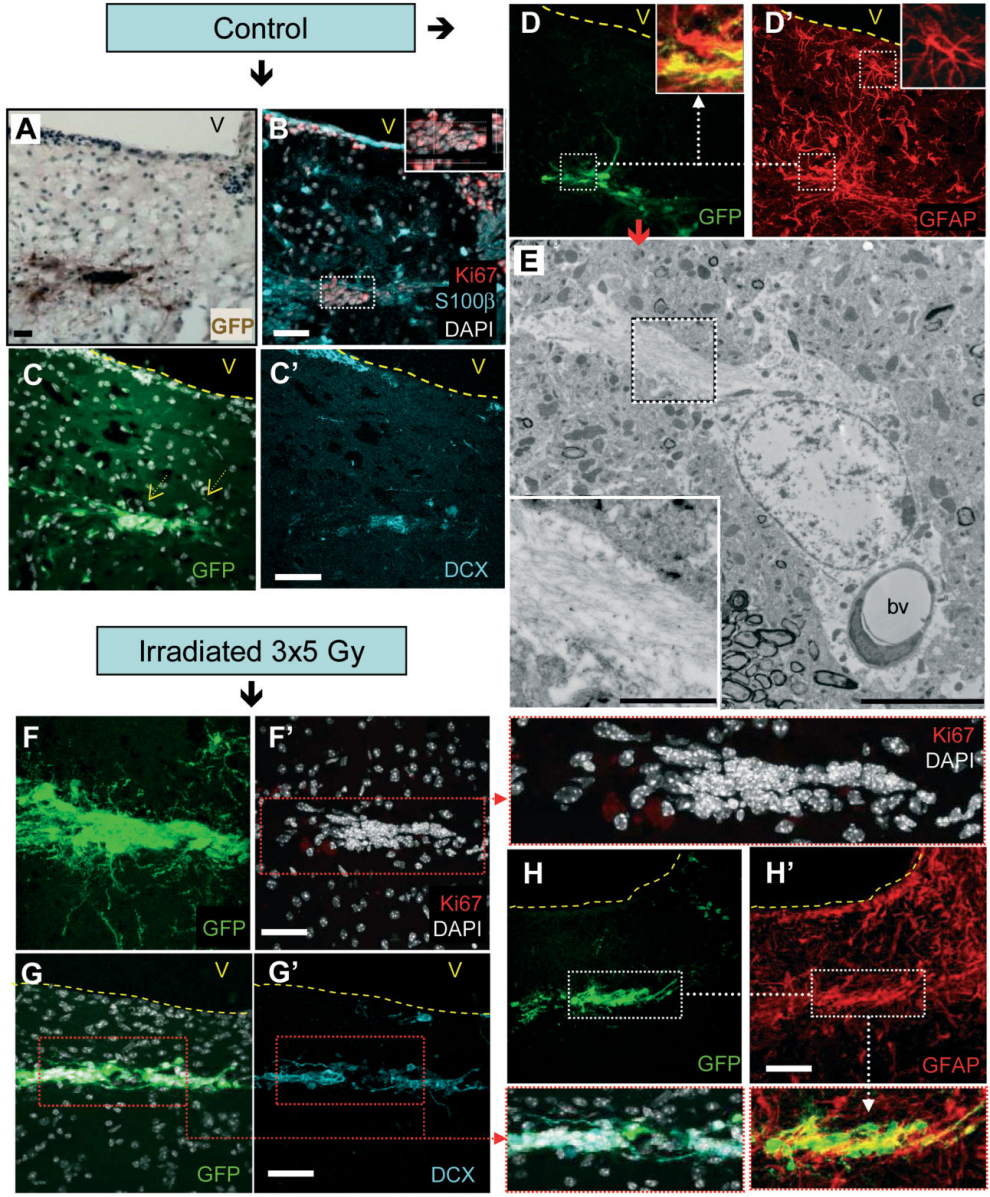
NSCs ceased to proliferate when grafted into irradiated SVZ niche. A–H. NSC/TAP-enriched SVZ cells from GFP mice were transplanted near to the SVZ in both control (A–E, *n* = 4) and irradiated mice (F–H′, *n* = 5). One month after grafting in non-irradiated hosts, the GFP grafts were composed of proliferating Ki67-positive cells (B, inset), Dcx^+^ neuroblasts (C and C′) and GFAP^+^ astrocyte-like cells (D and D′). GFP^+^ cells with an astrocyte-like phenotype had contact with blood vessels (E). The inset shows the GFP-immunogold labelling and the abundant filamentous components that are typical of astrocytes. When grafted into irradiated hosts, NSC/TAP-enriched GFP cells discontinue proliferation (F and F′) but still contain Dcx^+^ neuroblasts (G and G′) and GFAP^+^ cells (H and H′). Scale bars for light microscopy: 50 µm; for electron microscopy: 10 µm and inset 1 µm.

Antibodies to clusters of differentiation markers were used to remove endothelial cells (CD31), microglial/blood cells (CD45) as well as ependymal and neuroblasts (CD24) from young mouse SVZs. Freshly sorted CD24^−^CD31^−^CD45^−^ triple negative cells were enriched in neural stem/progenitor cells (58% GFAP^+^, 51% Sox2^+^ and 26% LeX^+^) and nearly devoid of neuroblasts (<1% Dcx^+^). NSC/TAP-enriched GFP^+^ cells were unilaterally transplanted near the SVZ in adult hosts. One month following transplantation, no evidence of graft rejection, such as phagocytosis by host cells, was observed (Supporting Information Fig S6A). GFP^+^ cells in the non-irradiated hosts were composed of proliferating Ki67-positive and Dcx-positive cells (Fig 2A,C and Table 1). A third of the GFP^+^ cells expressed GFAP, whereas these cells were negative for S100β, indicating that they were not mature astrocytes (Fig 2B,D and Table 1). Moreover, grafted GFAP^+^ cells exhibited a small number of elongated cytoplasmic processes that were similar to those of NSCs, distinguishing them from the typical stellate morphology of mature astrocytes. A subset of GFP^+^ cells were observed along the rostral migratory stream and integrated into the OBs, expressing neuronal-lineage markers, such as NeuN (Supporting Information Fig S6C). Moreover, GFP^+^ cells that had characteristics of functional neurons, with a dendritic spine that contacted the synapse of a host cell, were observed by electron microscopy (Supporting Information Fig S6D). These observations indicated that grafted GFP^+^CD24^−^CD31^−^CD45^−^ cells were neurogenic. Furthermore, it is worth noting that electron microscopic analyses of the graft demonstrated that GFP^+^ astrocyte-like cells were in close contact with blood vessels (Fig 2E), similar to what has been observed for normal endogenous type B NSCs (Tavazoie et al, 2008). Thus, these transplantation experiments recapitulated endogenous SVZ neurogenesis within vascular niches.

**Table 1:** Phenotype of grafted GFP cells after transplantation within SVZ

Mice (*n*)	GFP cells			
	
	Cells/graft	Ki67 (%)	GFAP (%)	Dcx (%)
Ctrl (4)	5959 ± 3090	32 ± 26	38 ± 23	50 ± 26
3 × 5 Gy (5)	4374 ± 1301	0 ± 0	26 ± 13	28 ± 5
	*p* = 0.274	*p* = 0.008	*p* = 0.200	*p* = 0.170

The *p*-value was calculated by Mann–Whitney *U*-test.

In sharp contrast, although NSC/TAP-enriched GFP^+^ cells survived for 1 month when grafted into the SVZ of irradiated hosts, they ceased proliferating, as was revealed by the absence of Ki-67 expression and the lack of BrdU incorporation (Fig 2F and Supporting Information Fig S6B). Nonetheless, a quarter of the GFP^+^ cells in the SVZ still expressed GFAP, whereas a third differentiated into Dcx^+^ neuroblasts (Fig 2G,H and Table 1). A subset of GFP^+^ cells integrated in the OBs and expressed neuronal markers (Supporting Information Fig S6C and D).

These transplantation data suggest that modifications of the vascular SVZ niche following irradiation are involved in the inhibition of NSC proliferation and the subsequent decrease in neurogenesis.

### Neither irradiation nor aging increase the mural coverage of SVZ microvessels

**Figure 3:**
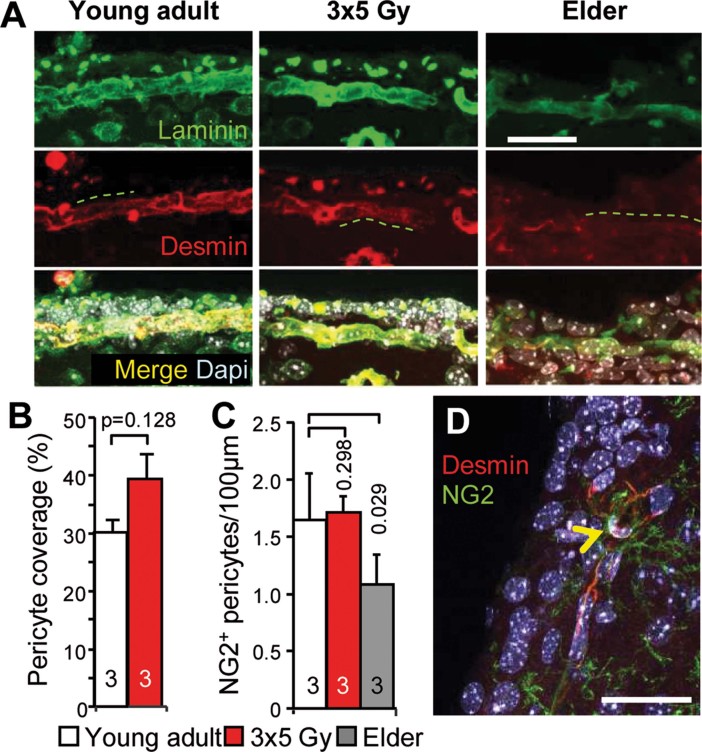
Pericyte coverage of SVZ capillaries is not increased following irradiation and during aging. Scale bars = 50 µm. The *p*-value was determined using the Mann–Whitney *U*-test (B) and the Kruskal–Wallis test (C). A. SVZ capillaries presented laminin immunostaining but had partial desmin mural coverage, with this marker being absent from regions with numerous nuclei (dashed line). B. The pericyte coverage of SVZ microvessels was examined using electron microscopy. C. The quantification of NG2/pericytes on SVZ capillaries following irradiation and during aging. The mean ± SD is shown and the number of mice is indicated within the bars. D. A NG2/pericyte on a SVZ capillary is shown (arrowhead).

One may speculate that irradiation-induced increases in the mural coverage of SVZ microvessels would disturb the intimate interactions of NSCs with endothelial cells and thereby impact neurogenesis. SVZ capillaries that were just beneath and tangential to the ependymal layer were analysed by immunostainings for laminin and desmin which are a major component of the basement membrane and an intermediate filament expressed on pericytes, respectively. In non-irradiated controls, the capillaries presented staining for laminin and partially covered with desmin (Fig 3A). Consistently, electron microscopy revealed that only 30% of the SVZ capillary surface were covered by pericytes (Fig 3B), which is in agreement with the partial coverage of SVZ microvessels (Tavazoie et al, 2008). NG2/desmin immunostainings revealed 1.5 pericytes per 100 µm of length of SVZ capillaries in the control mice (Fig 3C and D). Despite a slight increase in microvessels coverage (Fig 3B), irradiated mice did not present any significant effects on pericytes (Fig 3A–C).

The effects of aging on SVZ capillaries were somewhat different, as desmin immunostaining was diminished (Fig 3A) and a decrease was observed in the number of NG2/pericytes (Fig 3C).

Therefore, our data exclude the possibility that an increase in the mural coverage of SVZ blood vessels was involved in the decrease in neurogenesis following irradiation or during aging.

### TGF-β1 and Smad3 signalling increase in the vascular niche during aging and following irradiation

We hypothesized that factor(s) from SVZ endothelial cells may directly perturb NSCs. TGF-β1 is the prototypical anti-mitotic cytokine and that the cytostatic effects of TGF-β are critical for homeostasis in many epithelial tissues (Massague, 2012). Furthermore, this factor is upregulated in the brain after different forms of injuries (Gomes et al, 2005) and during aging (Werry et al, 2010).

**Figure 4:**
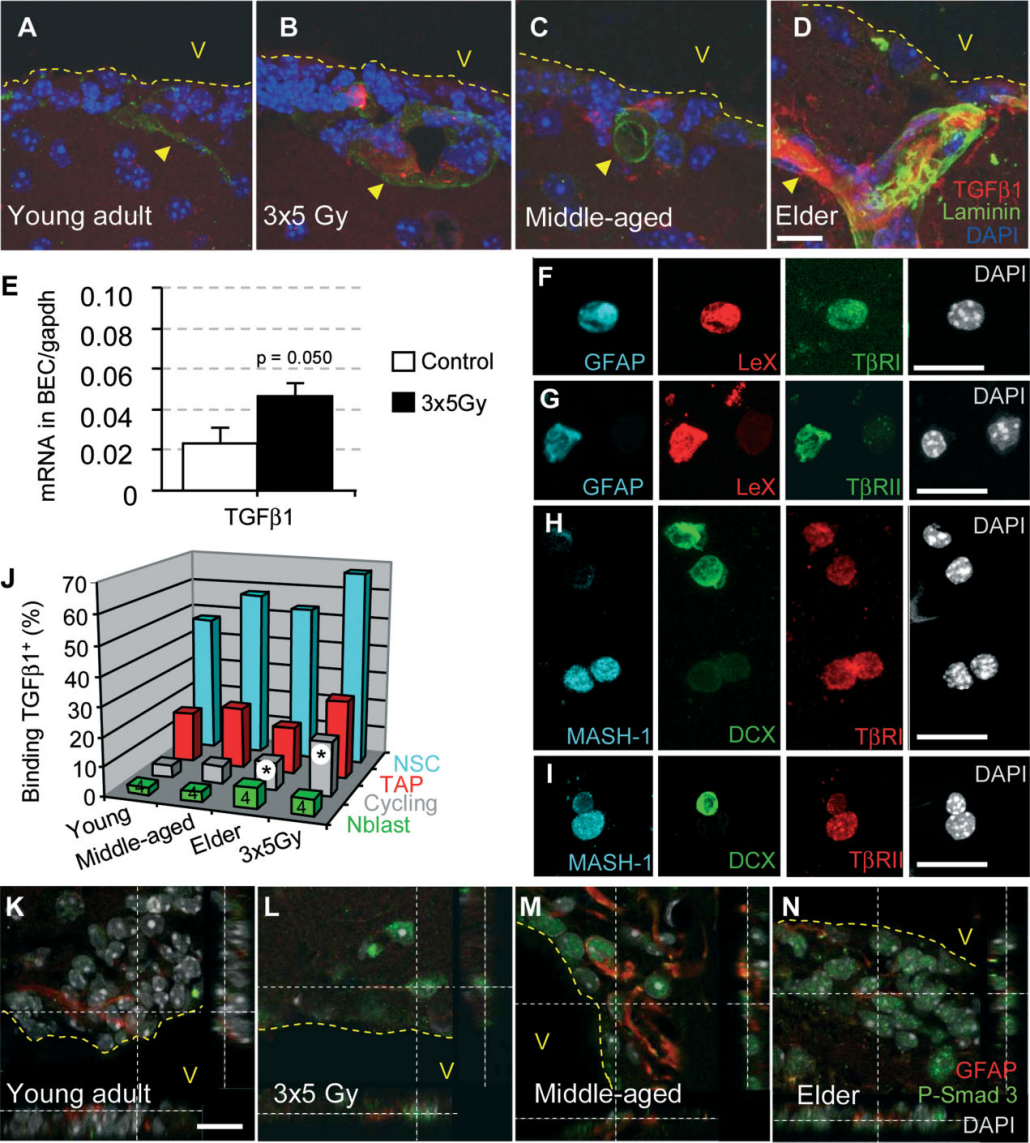
TGF-β/Smad3 signalling increases in the stem cell niche during aging and following irradiation. The data are represented as the mean ± SD The *p*-value was determined using the Mann–Whitney *U*-test (*p = 0.020; all of the other *p* values are given in Supporting Information Table 2). The illustrations are representative of three different experiments, with three mice per group. Scale bars = 10 µm. A–D. Increasing levels of TGF-β1 were observed in close contact with SVZ microvessels (laminin-positive) during aging and following irradiation. E. TGF-β1 mRNA expression by qPCR in sorted BECs. F–I. TβRI and TβRII are abundant on freshly dissociated GFAP^+^LeX^+^ and Mash1^+^ cells. J. The binding of biotinylated TGF-β1 to neuroblasts (CD24^+^), NSCs (GLAST^+^CD24^−^) and proliferating cells (DNA >2N). K–N. The phosphorylation of Smad3 was observed in the majority of SVZ cells in irradiated and aged mice, including cells with a GFAP^+^ type B/NSC phenotype.

We observed TGF-β1 immunostaining that was associated with microvessels in the SVZ from irradiated and middle-aged animals (Fig 4B and C), an effect that was remarkably stronger in elderly mice (Fig 4D). In contrast, staining was nearly undetectable in young adult mice (Fig 4A). To confirm that TGF-β1 was indeed upregulated in irradiated BECs, the cells were sorted by FACS using an anti-CD31 antibody from whole brains 4 months following irradiation. Blood cells were excluded using an anti-CD45 antibody. Quantitative RT-PCR analysis indicated that the expression level of TGF-β1 was increased by twofold in irradiated BECs (Fig 4E). We therefore conclude that TGF-β1 production increased in vascular niche BECs following irradiation and during aging.

Two TGF-β receptor chains, TβRI and TβRII, are required for TGF-β1 binding and signalling through Smad2/3 phosphorylation and for the latter's subsequent translocation into the nucleus (Massague, 2012). TβRII expression has been observed on nestin-positive adult neural progenitors (Wachs et al, 2006). We analysed the expression of TβRI and TβRII using immunofluorescence on freshly dissociated SVZ cells from control young adult mice. The expression of both receptors was associated with NSCs and TAPs (GFAP^+^LeX^+^ and Mash1^+^), whereas both receptors were scarcely expressed in Dcx^+^ neuroblasts (Fig 4F–I).

We further examined the binding of biotinylated TGF-β1 on SVZ cells from young mice and observed that it bound to nearly 50% of activated NSCs but to only 17% of TAPs and 3% of neuroblasts (Fig 4J and Supporting Information Table 1). This result confirmed the preferential expression of TGF-β receptors on NSCs. A significant increase in TGF-β1 binding was observed on cycling SVZ cells (DNA content >2N) during aging and following irradiation (Fig 4J).

Smad2 phosphorylation was undetected in the SVZ, even following irradiation or during aging (Supporting Information Fig S7). However, strong Smad3 phosphorylation was observed in the nucleus of the majority of SVZ cells from irradiated mice as well as in most SVZ cells from aged mice. In contrast, Smad3 staining was barely detectable in SVZ cells from young adult mice (Fig 4K–N). Interestingly, GFAP^+^ cells that were localized beneath the ependymal layer exhibited Smad3 phosphorylation, suggesting that the activation of TGF-β signalling occurred in NSCs following irradiation and aging (Fig 4L–N).

In conclusion, TGF-β production increased in vascular niches, particularly by BECs, during aging and following irradiation. This increased production in turn activated TGF-β/Smad3 signalling in NSCs and TAPs.

### Irradiated BECs provoke neural stem/progenitor apoptosis through TGF-β

**Figure 5:**
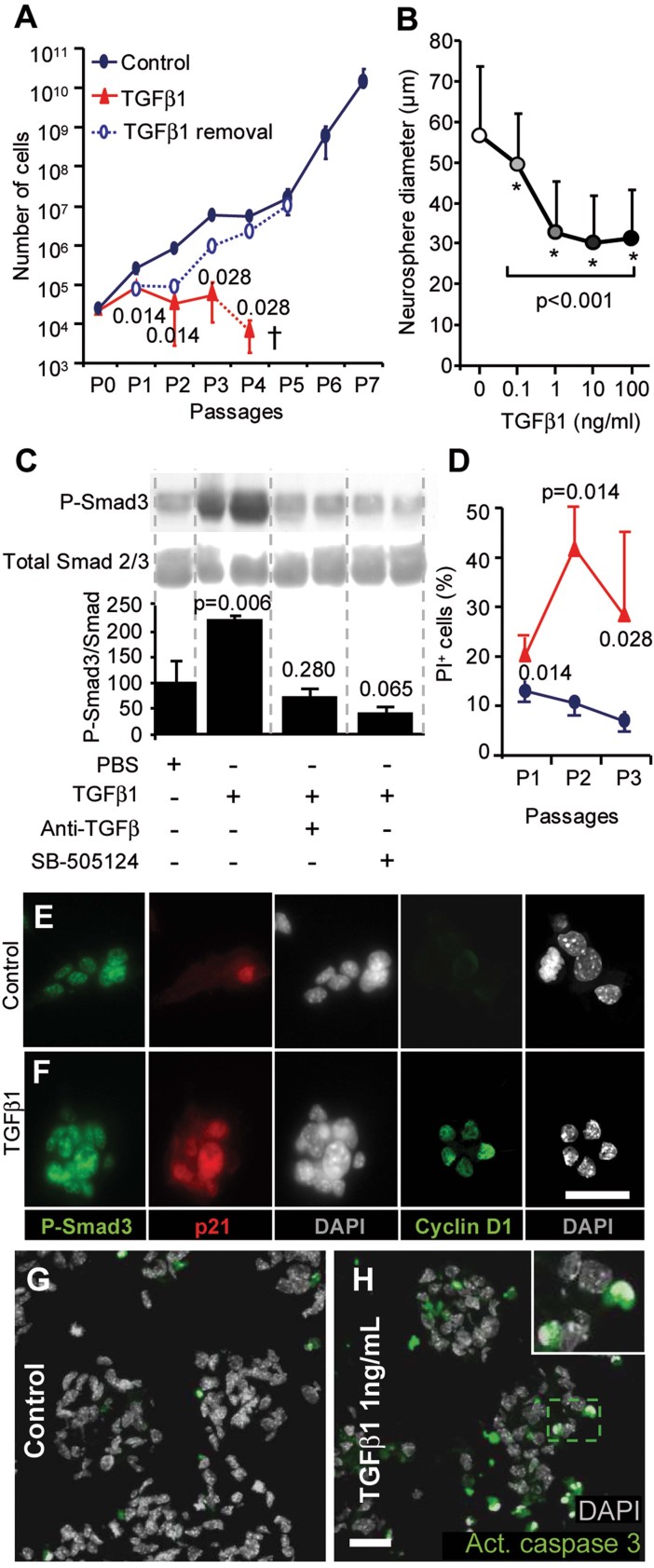
TGF-β signalling delays neurosphere growth and induces the apoptosis of neural stem/progenitor cells. The mean ± SD is shown. The *p*-value was determined using the Mann–Whitney *U*-test (A, C and D) and Student's *t*-test (B). Scale bars = 10 µm. A. Absolute number of cells in neurosphere cultures in the presence of TGF-β1 (10 ng/ml) at each passage (†cultures stopped at the fourth passage) or in which TGF-β1 was removed after the first passage. B. Neurosphere size 7 days following the addition of different concentrations of TGF-β1. C. The phosphorylation of Smad3 following the addition of TGF-β1, and the reversal of this effect by blocking with an anti-TGF-β antibody or SB-505124. The histogram represents the quantifications of the western blots, with normalization to total Smad2/3. Source data is available for this figure in the Supporting Information. D. Dying cells (permeable for propidium iodide) were quantified by FACS. E,F. P-Smad3, p21^*waf*^ and cyclin D1 were induced in neurospheres following TGF-β1 addition. G,H. Cleaved caspase 3 staining and pyknotic nuclei revealed apoptotic cells in neurospheres that were treated for 24 h with TGF-β1.

We analysed *in vitro* how TGF-β1 signalling affected the fate of neural stem/progenitor cells. Long-term neurosphere growth was clearly reduced upon the addition of 10 ng/ml of TGF-β1 in culture (Fig 5A). The transient addition of TGF-β1 during the first week reduced proliferation in a reversible manner; however, its continuous addition completely ceased growth at the fourth passage (Fig 5A). The size of neurospheres was significantly reduced (Fig 5B); however, the number of initiated neurospheres was unaltered, as previously reported (Wachs et al, 2006). This effect of TGF-β1 was dose-dependent, being maximal at 1 ng/ml, and was prevented by the addition of an anti-TGF-β blocking antibody (Fig 5B and Supporting Information Fig S8).

As expected, the addition of TGF-β1 to the culture induced the rapid phosphorylation of Smad3 in neurosphere cultures from young adult mice, and this phosphorylation was specifically blocked by the anti-TGF-β blocking antibody or SB-505124, which is a selective inhibitor of TβRI (Alk5) (Fig 5C). We also observed a rapid increase in the expression of both p21^*Waf*^ and cyclin D1 in nearly all of the neurosphere cells (Fig 5E–F), a result that is consistent with the anti-proliferative effect of TGF-β1 on foetal neural progenitors (Seoane et al, 2004).

Subsequently, we analysed whether the inhibition of neurosphere growth in the presence of TGF-β1 was linked to the death of neural progenitors. The number of dying cells (permeable to propidium iodide) dramatically increased in the presence of TGF-β1 in neurosphere cultures for several passages (Fig 5D). Moreover, the addition of TGF-β1 also induced the expression of cleaved caspase 3 in neurospheres, and this expression was associated with pyknotic nuclei (Fig 5G and H). These data indicated that exposure to TGF-β1 promoted apoptosis in proliferating neural stem/progenitor cells.

**Figure 6:**
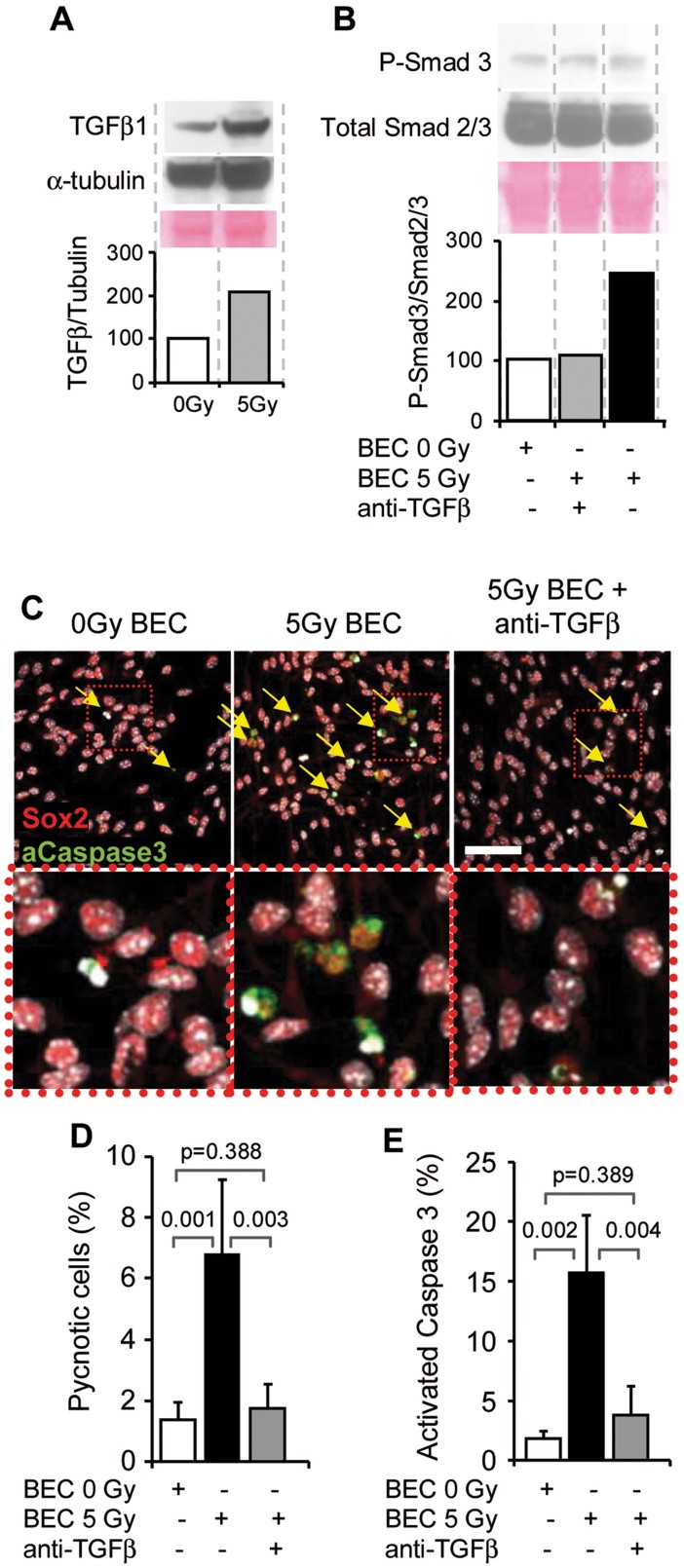
Irradiated BECs provoke neural progenitor apoptosis through TGF-β. Source data is available for this figure in the Supporting Information (A,B). A. The protein levels of TGF-β1 in BEC line (bEnd3) 24 h after 5 Gy irradiation. B–E. Irradiated or control neurosphere cells were co-cultured for 24 h in the presence of the BEC cell line with an anti-TGF-β antibody. (B) The level of P-Smad3 in neurosphere cells by western blot. (C–E) The apoptosis of neural stem/progenitor cells was quantified using cleaved caspase three staining and the counting of pyknotic nuclei. The mean ± SD from two independent experiments are represented in (D–E). The *p*-value was determined using the Kruskal–Wallis test. Scale bar = 50 µm.

Consistently with our *in vivo* data, 5 Gy radiation increased the levels of TGF-β1 in a BEC line (Fig 6A). We therefore performed co-culture experiments with irradiated BECs and neurosphere cells to model interactions within vascular SVZ niches. Neural progenitors that were obtained in neurosphere cultures were plated on laminin to allow for immunostaining of the isolated cells. Next, irradiated or non-irradiated BECs were added on top of the well that contained adherent neural progenitors. Sox2 and Mash1 were expressed in nearly all of the adherent cells in the co-culture, regardless of whether the BECs were irradiated (Fig 6C and Supporting Information Fig S9). This result confirms that they were indeed neural stem/progenitors, *i.e.* activated NSCs and TAPs. As anticipated, the phosphorylation of Smad3 increased in neural stem/progenitors in the presence of irradiated BECs compared to the control BECs in a TGF-β-dependent manner, as the addition of the anti-TGF-β blocking antibody abolished this increase (Fig 6B). The analyses of pyknotic nuclei and activated caspase 3 positivity revealed that a small number of apoptotic cells were observed in co-culture for 24 h with non-irradiated control BECs; however, the level of apoptotic neural stem/progenitors (*i.e.* Sox2^+^) significantly increased in the presence of irradiated BECs (Fig 6C–E). Remarkably, irradiation-induced apoptosis of neural progenitor was completely reversed with the addition of the anti-TGF-β neutralizing antibody (Fig 6C–E).

Our data indicate that the release of TGF-β by irradiated BECs promoted the apoptosis of NSCs and TAPs.

### The pharmacological blockade of TGF-β signalling restores neurogenesis *in vivo*

If TGF-β1 levels increase during aging or following irradiation, it appears logical to hypothesize that the blockade of TGF-β signalling in irradiated mice would enhance neurogenesis *in vivo*. Two different approaches were used to block TGF-β signalling *in vivo*. First, the intravenous administration of anti-TGF-β antibody was utilized given that the increase in TGF-β1 was associated with blood vessels. Second, SB-505124 was administered intranasally. Intranasal delivery is an efficient route for the transport of drugs into the central nervous system (Prediger et al, 2010); moreover, this type of delivery eliminates the undesired side effects that are characteristic of systemic delivery. The treatments were administered three times over 5 days in irradiated or elderly mice.

The effects of blockade of TGF-β signalling were first examined on SVZ capillaries, as this cytokine is known to exert different effects on vascular development, including the induction of pericyte differentiation and adhesion, as well as the production of extracellular matrix or basement membrane (Winkler et al, 2011). Both the anti-TGF-β antibody and SB-505124 dramatically reduced the levels of laminin and decreased desmin coverage in irradiated and aged mice (Supporting Information Fig S10A). However, the treatments did not alter the number of NG2/pericytes (Supporting Information Fig S10B).

**Figure 7:**
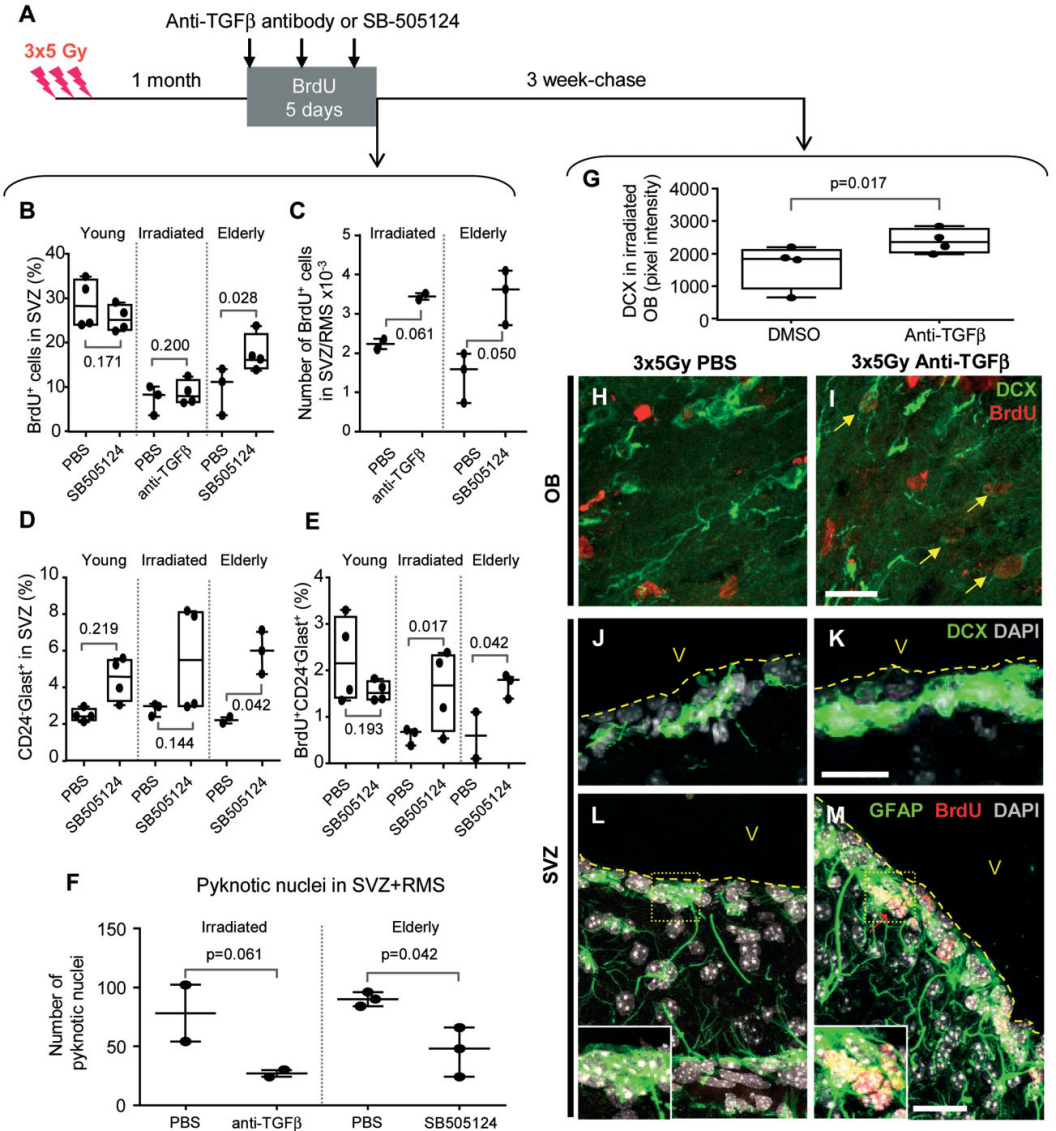
Pharmacological blockade of TGF-β signalling restores neurogenesis *in vivo.* The mean value for each mouse is plotted individually, and the error bars represent the standard deviation that was obtained from two independent experiments. The *p*-value for comparison between treated and control groups was determined using the Mann–Whitney *U*-test (F–G). A. Experimental schedule for the treatment by intravenous injection of anti-TGF-β–neutralizing antibody or the intranasal administration of SB-505124. The mice were euthanized 1 day after the final treatment (left panel) or after a 3-week chase (right panel). B,C. The quantification of BrdU incorporation in SVZ cells by FACS (B) and by immunostaining (C). D,E. FACS analysis of NSCs (D) and BrdU incorporation in NSCs (E) following SB-505124 treatment in old or irradiated mice. F. The quantification of pyknotic nuclei in serial coronal SVZ sections. G–M. The irradiated mice were treated with an anti-TGF-β antibody (H, J, L) or untreated (I, K, M). In the OBs, anti-TGF-β antibody treatment increased the Dcx fluorescence intensity (G) and the number of BrdU/Dcx-double-positive neuroblasts (arrows in I). In the SVZ, anti-TGF-β antibody treatment increased Dcx staining (K) and the presence of candidate NSCs GFAP^+^ with long-term BrdU label retaining (M, red arrow). Scale bar = 50 µm.

Next, BrdU incorporation was used to examine the effects of anti-TGF-β therapy on neurogenesis. This analysis was performed to estimate cell proliferation during the treatment as well as to trace newly generated cells (Fig 7A). To obtain quantitative data for the entire SVZ, BrdU incorporation was first examined by FACS. As expected from the decrease in neurogenesis, BrdU incorporation was noticeably lower in SVZ cells from irradiated mice as well as in SVZ of elderly mice compared to young adult mice (Fig 7B, *p* = 0.025 and *p* = 0.050, respectively). The blockade of TGF-β signalling in irradiated and aged mice, using the anti-TGF-β neutralizing antibody and SB-505124 increased the number of proliferating cells in the SVZ (Fig 7B). This increase in the number of BrdU^+^ cells in the SVZ of irradiated and aged mice receiving anti-TGF-β therapy was further confirmed by microscopic analyses (Fig 7C). By contrast, the treatment of young adult mice with SB-505124 did not alter the number of BrdU^+^ cells (Fig 7B), suggesting that the efficacy of anti-TGF-β signalling was only related to the elevated TGF-β levels that were observed in the pathophysiological conditions of aging and radiation exposure.

The amelioration of the decline in neurogenesis was also demonstrated by an increase in neuroblast production in the SVZ in both irradiated and aged mice receiving anti-TGF-β therapy (Supporting Information Fig S11A). We then traced the fate of new neuroblasts 3 weeks after BrdU incorporation, which is sufficient time for them to reach the OBs. Dcx immunostaining in the OBs increased in irradiated mice 3 weeks following treatment with the anti-TGF-β antibody in comparison to irradiated mice (Fig 7G), confirming the efficacy of anti-TGF-β therapy for improving neurogenesis. Moreover, numerous Dcx^+^ cells in the OBs were also BrdU-positive (Fig 7H and I), strongly suggesting that they originated from NSCs that had proliferated at the time of anti-TGF-β therapy. Interestingly, Dcx immunostaining remained elevated in the SVZ 3 weeks following treatment (Fig 7J and K), indicating that anti-TGF-β therapy had long-lasting effect on neurogenesis and most likely targeted immature neural stem/progenitor cells. Remarkably, numerous GFAP^+^ cells lining the lateral ventricle retained BrdU labelling long-term following the administration of SB-505124 in irradiated mice (Fig 7M), indicating that anti-TGF-β treatment increased the number of NSCs with long cell cycle (Morshead et al, 1998). We then quantified BrdU incorporation by FACS in candidate NSCs with the CD24^−^GLAST^+^ phenotype. The level of CD24^−^GLAST^+^ cells was unaltered in irradiated and aged mice compared to young adults (Fig 7D); however, their proliferation rate (BrdU incorporation) was diminished (Fig 7E), confirming the data that are shown in Fig 1F′. The administration of SB-505124 slightly increased the percentage of CD24^−^GLAST^+^ cells in the SVZ, an effect that reached statistical significance for aged mice (Fig 7D). Furthermore, the treatment of irradiated or aged mice with SB-505124 significantly increased the BrdU incorporation within CD24^−^GLAST^+^ cells, indicating that this treatment provoked cell cycle entry of NSCs; however, this treatment had no effect in young adult mice (Fig 7E). These results were further confirmed by FACS analyses on CD24^−^LeX^+^ NSCs (Supporting Information Fig S11).

We also examined the effect of anti-TGF-β therapy on apoptosis by estimating the number of pyknotic nuclei for the entire SVZ and RMS. The administration of the anti-TGF-β antibody to irradiated mice reduced the number of apoptotic cells; similar results were observed in aged mice following treatment with SB-505124 (Fig 7F).

Together, these experiments demonstrated that the selective blockade of TGF-β signalling improved neurogenesis in aged and irradiated mice by preventing the apoptosis of neural progenitors and by inducing the proliferation of NSCs.

## DISCUSSION

Our study concludes that, although neurogenesis decreased during aging and following high-dose radiation, many NSCs resisted high radiation doses and survived for several months while retaining their stemness characteristics. Our data indicate that the NSC niche becomes functionally impaired following radiation exposure and during aging. Both of these causes increase TGF-β1 levels in BECs, causing the quiescence and apoptosis of NSCs via a TGF-β/Smad3 dependent pathway. Interestingly, the inhibition of TGF-β signalling allows for neurogenesis to efficiently recover following irradiation, suggesting that it may have far-reaching implications for aged individuals and for patients with cancer who are treated with cranial radiotherapy.

### The perturbation of the vascular niche during aging and following irradiation

The vasculature is a key component of the adult SVZ NSC niche with respect to the proliferation of NSCs and TAPs under physiological conditions (Tavazoie et al, 2008). Our results illustrate that the alteration of neurogenesis that occurs following irradiation is primarily due to modifications of the vascular niche. Indeed, grafted astrocytic-like NSCs extend contacts toward brain microvascular BECs, similar to endogenous NSCs (Tavazoie et al, 2008); however, these cells stop proliferating when transplanted into irradiated hosts. We further demonstrated that an increase in the mural coverage of SVZ blood vessels was not involved in the neurogenesis decline following irradiation or during aging.

The mobilization and activation of microglial cells into the hippocampus have been implicated in the inhibition of neurogenesis following the administration of a single 10 Gy irradiation, and the blockade of inflammation restores neurogenesis (Monje et al, 2003). In contrast, in the present study, we did not detect any excess of microglial cells in the SVZ following irradiation. The discrepancy between our study and that of Monje et al (2003) may be attributable to the irradiation protocol, given that a split dose of 15 Gy may induce less disruption to the blood brain barrier than a single 10 Gy dose (Trnovec et al, 1990).

We also demonstrate that BECs synthesize more TGF-β1 as they are irradiated and that the increase in the TGF-β1 levels within vascular niches is an early molecular sign of aging in middle-aged mice. Other mechanisms, such as the activation of the latent TGF-β complex with integrins or the release of circulating TGF-β1 owing to blood–brain barrier breakdown, may contribute to increase in the TGF-β1 levels within vascular neurogenic niches following radiation exposure or during aging. Our data are interesting with regards to the increase in TGF-β1 that is reported in the human brain during aging (Werry et al, 2010).

The augmentation of TGF-β1 in the SVZ vascular niche during aging and following irradiation is associated with the activation of the canonical TGF-β signalling pathway in SVZ cells, including NSCs. Thus, TGF-β1 overproduction by BECs following irradiation and during aging may participate in the deregulation of neurogenesis.

### Mechanism of neurogenesis perturbation by TGF-β1

Here, we unambiguously demonstrate that NSCs and TAPs are primary targets for TGF-β1, and an increase in TGF-β1 levels during aging and following irradiation leads to the inhibition of neurogenesis. We demonstrate that TβR chains are present on both NSCs and TAPs. Furthermore, we demonstrate that TGF-β1 binding increases with aging and following irradiation. From a mechanistic perspective, we demonstrate that the activation of the canonical TGF-β signalling pathway via the phosphorylation of Smad3, but not of Smad2, occurs in NSCs and TAPs *in vivo* and *in vitro*. Although we demonstrated that TGF-β/Smad3 signalling is activated in neural stem/progenitors, a genetic approach, such as TβR inactivation in neural stem/progenitors, would be necessary to exclude the possibility that TGF-β plays an indirect role, *e.g.* through the microenvironment. Whereas Smad3 triggers TGF-β, activin and Nodal signalling, our data suggest that Smad3 is activated in response to TGF-β given that its phosphorylation is specifically blocked with the anti-TGF-β antibody.

Previous studies reported the negative effects of TGF-β1 on adult neurogenesis and neural progenitor proliferation in both the hippocampus and the SVZ (Buckwalter et al, 2006; Wachs et al, 2006). It has also been observed to have apoptotic effects on proliferating neural-crest-derived multipotent progenitor cells (Hagedorn et al, 2000). Our data demonstrate that TGF-β1 induces apoptosis of proliferating Mash1^+^/Sox2^+^ neural stem/progenitors.

The increased expression of cyclin D1 stimulated by TGF-β1 may participate in initiating apoptosis in neural stem/progenitors, as has been reported for other cell types (Han et al, 1996). Notably, we also demonstrate that co-culturing with irradiated BECs induces the apoptosis of neural stem/progenitors in a TGF-β-dependent manner, underscoring the importance of BECs in the radiation-induced decline of neurogenesis. Remarkably, anti-TGF-β therapy, using either a blocking antibody or a selective TβR inhibitor, efficiently reduces apoptosis and enables neurogenesis to recover in aged and irradiated mice. Therefore, the apoptosis of proliferating cells in the SVZ following irradiation and during aging is a characteristic of the action of TGF-β1 on proliferating NSCs and TAPs. We cannot make conclusions regarding possible differences in sensitivity to TGF-β between activated NSCs and TAPs; however, the persistence of quiescent NSCs in irradiated or aged mice indicate that TGF-β has no or limited effects on the viability of these cells.

A transition of activated NSCs to a quiescent state rather than a loss of NSCs occurs during aging in the hippocampus is involved in neurogenesis decline (Lugert et al, 2010). Our data also support the conclusion that NSCs entered dormancy in the SVZ, an effect that is due to elevated TGF-β signalling in the vascular niche. Indeed, *ex vivo* FACS analyses clearly indicate that NSCs are more quiescent following irradiation or during aging. Treatment with anti-TGF-β therapy using either a blocking antibody or a TβR inhibitor induces proliferation in the SVZ of aged and irradiated mice. Strikingly, treatment with anti-TGF-β therapy promotes NSCs to enter the cell cycle (as shown by increases in CD24^−^GLAST^+^BrdU^+^ and CD24^−^LeX^+^BrdU^+^ cell numbers) in irradiated and aged mice. The fact that anti-TGF-β therapy has long-lasting effects on neuroblast production in the SVZ confirms that it primarily targets immature cells, *i.e.* NSCs. However, blocking TGF-β signalling in young adult mice, *i.e.* prior to the point at which an increase in TGF-β signalling is observed, does not alter the number of BrdU^+^ cells or cell cycle entry of NSCs, suggesting that the decrease in neurogenesis by TGF-β1 is only related to the pathophysiological conditions of aging and radiation exposure.

We therefore report on a novel mechanism of neurogenesis decline following irradiation and during aging, one that perturbs the vascular niche via the upregulation of TGF-β, resulting in NSC quiescence and the apoptosis of proliferating neural stem/progenitor cells.

### The blockade of TGF-β signalling in radiotherapy and aging

The split dose of 15 Gy delivered over three sessions is clinically relevant to prophylactic cranial radiation for brain metastasis, and it is well below the threshold for the vascular damage and white matter necrosis that have been observed long-term in the mouse brain following radiation exposure (Calvo et al, 1988). However, this irradiation regimen induces olfactory memory deficits in mice (Lazarini et al, 2009). The long-term effects of irradiation on normal tissues are a major limitation in increasing the dose for the eradication of cancer cells. That is, preventing or reducing the long-term side effects of irradiation has increasingly become a priority in the improvement of both tumour treatments and outcomes for patients with cancer. An increase in TGF-β1 levels is involved in the well-documented long-term side effects of radiotherapy, *e.g.* fibrosis of the kidney, skin, lungs and intestine. Autocrine TGF-β signalling maintains the tumourigenicity of glioma-initiating cells (Seoane, 2009). The development of TGF-β signalling inhibitors has thus become an object of study in cancer therapy fields (Yingling et al, 2004).

Future research should determine whether the new neurons that are produced in the presence of anti-TGF-β therapy are functional and if our results can be extended to the hippocampus, where NSCs niche near to the vasculature.

Based on these findings, it appears a worthwhile goal to evaluate the efficacy of anti-TGF-β therapy to treat radiotherapy-induced ionising radiation injury or to rejuvenate neurogenesis in aged individuals who exhibit cognitive decline.

## MATERIALS AND METHODS

### Animals and irradiation procedure

C57Bl/6 and actin-GFP (Okabe et al, 1997) mouse strains were used in the present study. Mice of different ages were used: young adult (3–6 months), middle-aged (10–14 months) and elderly (15–24 months). The animals were maintained with access to food and water ad libitum in a colony room that was maintained at a constant temperature (19–22°C) and humidity (40–50%) on a 12:12 h light/dark cycle.

The heads of 2-month-old male C57Bl6J mice were exposed to a ^60^Co source with a medical irradiator (Alcyon) while under ketamine- (75 mg/kg; Imalgen, Merial, France) and medetomidine-induced anaesthesia (1 mg/kg; Domitor, Pfizer, France). One lead shield protected the body of the mouse during exposure. A total dose of 15 Gy was given at a dose rate of 1 Gy/min in three equal fractions that were separated by 48 h intervals (Lazarini et al, 2009). After exposure, the mice were woken up via an i.p. injection of atipamezole (1 mg/kg; Antisedan, Pfizer, France).

The animal experiments were performed in compliance with the European Communities Council Directive of November 24, 1986 (86/609/EEC) and were approved by our institutional committee on animal welfare (CETEA-CEA DSV IdF).

### Drug administration

During the 5 days of drug administration, the mice received BrdU (Sigma) in their drinking water at a concentration of 1 mg/L. The mice were treated three times on Days 1, 3 and 4 with a neutralizing antibody that recognizes all TGF-β-1, -2, -3 forms (mAb1835; R&D) or a selective TβRI inhibitor, SB-505124 (Sigma). Fifty microgrammes of anti-TGF-β neutralizing antibody that was diluted in 50 µl of NaCl 0.9% were administered intravenously under ketamine/medetomidine-induced anaesthesia. Ten microlitres of SB-505124 (360 µM diluted in saline) were administered per nostril using a cannula with an inner diameter of 0.2 mm that was adapted to a 10 µl Hamilton syringe and a nano-injector (KD Scientific, Holliston, MA) at a dose-rate of 12 µl/min, as previously described (Prediger et al, 2010).

### Flow cytometry analyses and cell sorting

The SVZ microdissection, papain dissociation and staining with the vital DNA dye Hoechst 33342 (1 µg/ml for 1 h 30 min) were performed following previously described methods (Mouthon et al, 2006). For BEC isolation, C57Bl/6 brains were dissociated in collagen, as previously reported (Mathieu et al, 2008). The SVZ cells were incubated for 15 min in PBS 0.15% BSA at 4°C with the following fluorescent-coupled antibodies: CD24-PE, LeX/CD15-FITC, CD31-PE, CD45-PC5/PE and GLAST-APC, or with EGF-Alexa647 (1:200; Invitrogen).

For the TGF-β-binding experiments, freshly dissociated SVZ cells were labelled using human TGF-β1 biotinylated/avidin-FITC Fluorokine Kit (R&D). For the BrdU staining, the cells were fixed with Cytofix/Cytoperm and processed using a BrdU Flow kit, as recommended by the manufacturer (BD). The DNA content was assessed using 7-AAD staining.

The cells were sorted on a FACS (INFLUX cell sorter, BD) at a pressure of 20–40 psi or analysed using a LSR II SORP flow cytometer (BD). Doublets were excluded on the basis of DNA dye fluorescence in the Height *versus* Wide graph. The FACS data were analysed using FlowJo software (Version 7.2.5).

**The paper explained**

**PROBLEM**

A progressive cognitive decline occurs during aging and following cranial radiotherapy and is currently untreatable. A key element of this decline is the decreased production of new neurons by neural stem cells. However, mechanisms that underlie changes that occur in both stem cells and neurogenesis are poorly understood.

**RESULTS**

One of our major findings is that a deficit in neurogenesis that is observed following high-dose radiation and during aging is due to alterations in the microenvironment that regulates stem cell fate rather than to a direct effect on the stem cells. Moreover, we have elucidated the molecular mechanism by which the increased synthesis of TGF-β1 by brain endothelial cells in the stem cell niche provokes stem cell dormancy and increases their susceptibility to apoptosis.

The second important finding is that the pharmacological blockade of TGF-β signalling restored the production of new neurons and their integration into the olfactory bulbs of irradiated and elderly mice.

**IMPACT**

Our newly discovered mechanism should encourage the development of anti-TGF-β therapies for (i) limiting brain injury that is caused by radiotherapy or (ii) rejuvenating neurogenesis in elderly individuals with cognitive decline.

### Transplantation

CD24^−^CD31^−^CD45^−^GFP^+^ cells from the SVZs of GFP^+^ mice were sorted using FACS. These GFP^+^ cells formed neurospheres in the presence of EGF and FGF2. Immediately after FACS, 1 µl of PBS that contained 10^4^ of CD24^−^CD31^−^CD45^−^GFP^+^ cells was administered transcranially at the following coordinates: AP = +0.75 mm, *L* = −1.1 mm and DV = −2.7 mm into anaesthetized C57Bl6J mice. The transplantations were performed using a small animal stereotaxic apparatus (Kopf model 900) with a 10 µl Hamilton syringe and a 33 G needle (Hamilton, Bonaduz, Switzerland).

### N-CFCA, neurosphere and adherent cultures

The neural colony-forming cell assays (NCFC-A; StemCell Technologies) were performed using freshly isolated SVZ cells, according to the manufacturer's instructions. After 21 days *in vitro*, the colonies were scored according to their size using an eyepiece reticule on an inverted light microscope under phase-contrast optics. Large (diameter ≥1.5 mm) and small clones (diameter <1.5 mm) were considered to have derived from NSCs and TAPs, respectively (Louis et al, 2008).

For the neurosphere cultures, freshly dissociated SVZ cells were plated in 6- or 12-well-plates with Neurocult complete medium (StemCell) that was supplemented with heparin (2 µg/ml), EGF (20 ng/ml; Invitrogen) and FGF2 (10 ng/ml; Invitrogen). To test the effects of TGF-β1 (1 ng/ml; R&D), it was added weekly at the time of passage.

For the adherent cultures, freshly dissociated SVZ cells or neurosphere cells were plated on poly-D-lysine- (Sigma) or laminin- (Sigma) coated flasks. The cultures were incubated in a humidified atmosphere with 5% CO_2_. The medium and growth factors were refreshed every 7 days.

### Brain endothelial cells and co-cultures

Two sources of adult mouse BECs were used: primary CD31^+^CD45^−^ BECs for the qRT-PCR analyses (described in Cell sorting), and the bEnd3 cell line for the co-cultures. The bEnd3 cell line originated from adult mouse BECs and was obtained from ATGC (CRL#2299). These cells were grown to a subconfluent monolayer in DMEM:F12 that was supplemented with 10% foetal calf serum. BEC monolayers were established 3–4 days before co-culture by plating 125 × 10^3^ cells on 0.5% gelatine-coated Transwells. The BECs were irradiated at a dose of 5 Gy (0.6 Gy/min) in Transwells. Following exposure, the media was rinsed and the BECs were placed on the top of an adherent NSC culture in Neurocult complete medium.

### Immunofluorescence and immunohistochemistry

Deeply anaesthetized animals received an intra-cardiac perfusion of 4% paraformaldehyde in 0.1 M sodium phosphate (pH 7.4). The brains were post-fixed for 2 h in 4% PFA and cryoprotected in an incremental 10–30% sucrose/PBS gradient. Serial coronal cryostat sections were made (10 µm for graft, and 30 µm treatment experiments). The sections were incubated for 1 h in PBS with 0.3% Triton-X100–1% BSA or in PBS with 0.1% Tween20 (for Mash-1). The sections were then incubated overnight at 4°C with primary antibodies (see Supporting Information Table 3 for the list of the primary antibodies). As it was difficult to localize TGF-β receptors *in situ*, immunofluorescences for TβRs were performed on freshly dissociated SVZ cells. After three washes in PBS, the sections were incubated with AlexaFluor donkey secondary antibodies at 1:200 (Millipore). The sections were rinsed and mounted with DAPI Fluoromount-G (Southern Biotech).

### The quantification of immunofluorescence

The immunofluorescence images were taken with 20× and 40× objectives using a Leica TCS SPE confocal microscope (Leica Application Suite, Leica Microsystems). Mosaic fluorescence images were obtained using a motorized microscope (Pathfinder, Imstar S.A., France) that was equipped with an Hamamatsu C8484-05G camera (Hamamatsu photonics, France) with NIS Elements software v3.1 (Nikon instruments, USA). For the BrdU and pyknotic nuclei analysis, the positive cells were counted from four to five consecutive sections that were separated by 140 µm along the entire SVZ. The results are presented as an estimate of the total number of positive cells per SVZ taking into the consideration the 140 µm spacing. The quantification of Dcx in OBs was performed by measuring fluorescence intensity using ImageJ software within a surface area that encompassed the RMS-OB.

### Ultrastructural analysis of the SVZ

Deeply anaesthetized animals received an intra-cardiac perfusion with 4% PFA in 0.1 M sodium phosphate (pH 7.4) with 0.5% glutaraldehyde. After post-fixation overnight at 4°C, the brains were embedded in agarose and then cut into 50 µm sections using a VT 1200S vibratome (Leica, France). The sections that contained grafted GFP^+^ cells were selected using a LUMAR v1.2 stereoscope (Zeiss, France). Pre-embedding and GFP-immunogold staining were performed as described (Sirerol-Piquer et al, 2012). The sections were contrasted with 1% osmium and 7% glucose and embedded in araldite. Semi-thin 1.5-µm sections were prepared, selected at the light microscope level, and re-embedded for ultrathin sectioning at 70 nm. The sections were examined and photographed under a transmission electron microscope (FEI Tecnai, Hillsboro, OR, USA) using a digital camera (Morada, Soft Imaging System). The different SVZ cell types and the coverage of blood vessels with pericytes were quantified on 60–70 nm ultrathin sections that were stained with lead citrate and were examined under a transmission electron microscope. The different cell types within the SVZ, *i.e.* adjacent to the ventricle lumen, were counted at three different rostro-caudal levels (0–1 mm anterior to bregma). The coverage of 10 blood vessels per section within the SVZ was measured using UTHSCSA ImageTool software and expressed as the percentage of blood vessels perimeter covered by pericytes.

### Western blots

The cells in the neurospheres or in the BEC co-cultures were harvested in lysis buffer (1% NP40, 20 mM Tris pH7.5, 150 mM NaCl, 10 mM glycerophosphate, 2 mM EGTA, 5 mM NaF and 1 mM sodium pyrophosphate) that was supplemented with Halt phosphatase inhibitor cocktail (Thermo Scientific). The extracts were vortexed for 1 min and centrifuged at 20 000*g* for 10 min at 4°C. The soluble fraction was used for electrophoresis and immunoblotting. After spectrometric protein quantification, 40 µg of protein was loaded on a 10% acrylamide gel. Electroblotting was performed at 30 V overnight. The membranes were blocked in 5% BSA/TBST buffer (20 mM Tris–HCl, 0.15 M NaCl, 0.1% Tween-20). The primary antibodies used are listed in the Supporting Information Table 3. The secondary HRP-conjugated goat anti-mouse or anti-rabbit antibodies (GE HealthCare) were used at 1:5000. Next, the membranes were subjected to chemioluminescence detection using SuperSignalWest Pico Chemiluminescent Substrate (Pierce, Erembodegem, Belgium).

### Quantitative PCR analysis

Total RNA was extracted from the sorted cells or total SVZ using Micro RNeasy plus isolation kits (Qiagen) and then reverse-transcribed using a high-capacity reverse transcription kit (Applied Biosystems). Q-PCR was performed on an ABI PRISM 7200 Sequence Detector System using SYBR Green PCR Master Mix (Applied Biosystems); the specific primers are listed in Supporting Information Table 4. Each sample was normalized to endogenous Gapdh expression. Confirmation was also obtained from normalization with 18S. Each reaction was performed at least twice in duplicate.

## Author contributions

MAM and FDB conceived the study. MAM and JRP designed experiments. The majority of the experiments were performed by JRP, with important contributions of MD, AC, KSF and MAM. JRP, MAM and FDB analysed the data. ACS and JMGV performed and interpreted the ultrastructural study. JRP, FDB and MAM wrote the manuscript.

## Notes

As a service to our authors and readers, this journal provides supporting information supplied by the authors. Such materials are peer reviewed and may be re-organized for online delivery, but are not copy-edited or typeset. Technical support issues arising from supporting information (other than missing files) should be addressed to the authors.

## References

[b1] Achanta P, Capilla-Gonzalez V, Purger D, Reyes J, Sailor K, Song H, Garcia-Verdugo JM, Gonzalez-Perez O, Ford E, Quinones-Hinojosa A (2012). Subventricular zone localized irradiation affects the generation of proliferating neural precursor cells and the migration of neuroblasts. Stem Cells.

[b2] Ahlenius H, Visan V, Kokaia M, Lindvall O, Kokaia Z (2009). Neural stem and progenitor cells retain their potential for proliferation and differentiation into functional neurons despite lower number in aged brain. J Neurosci.

[b3] Alvarez-Buylla A, Lim DA (2004). For the long run: maintaining germinal niches in the adult brain. Neuron.

[b4] Battista D, Ferrari CC, Gage FH, Pitossi FJ (2006). Neurogenic niche modulation by activated microglia: transforming growth factor beta increases neurogenesis in the adult dentate gyrus. Eur J Neurosci.

[b5] Boche D, Cunningham C, Gauldie J, Perry VH (2003). Transforming growth factor-beta 1-mediated neuroprotection against excitotoxic injury in vivo. J Cereb Blood Flow Metab.

[b6] Buckwalter MS, Yamane M, Coleman BS, Ormerod BK, Chin JT, Palmer T, Wyss-Coray T (2006). Chronically increased transforming growth factor-beta1 strongly inhibits hippocampal neurogenesis in aged mice. Am J Pathol.

[b7] Calvo CF, Fontaine RH, Soueid J, Tammela T, Makinen T, Alfaro-Cervello C, Bonnaud F, Miguez A, Benhaim L, Xu Y (2011). Vascular endothelial growth factor receptor 3 directly regulates murine neurogenesis. Genes Dev.

[b8] Calvo W, Hopewell JW, Reinhold HS, Yeung TK (1988). Time- and dose-related changes in the white matter of the rat brain after single doses of X rays. Br J Radiol.

[b9] Capela A, Temple S (2002). LeX/ssea-1 is expressed by adult mouse CNS stem cells, identifying them as nonependymal. Neuron.

[b10] Doetsch F, Caille I, Lim DA, Garcia-Verdugo JM, Alvarez-Buylla A (1999). Subventricular zone astrocytes are neural stem cells in the adult mammalian brain. Cell.

[b11] Enwere E, Shingo T, Gregg C, Fujikawa H, Ohta S, Weiss S (2004). Aging results in reduced epidermal growth factor receptor signaling, diminished olfactory neurogenesis, and deficits in fine olfactory discrimination. J Neurosci.

[b12] Gomes FC, Sousa Vde O, Romao L (2005). Emerging roles for TGF-beta1 in nervous system development. Int J Dev Neurosci.

[b13] Hagedorn L, Floris J, Suter U, Sommer L (2000). Autonomic neurogenesis and apoptosis are alternative fates of progenitor cell communities induced by TGFbeta. Dev Biol.

[b14] Han EK, Begemann M, Sgambato A, Soh JW, Doki Y, Xing WQ, Liu W, Weinstein IB (1996). Increased expression of cyclin D1 in a murine mammary epithelial cell line induces p27kip1, inhibits growth, and enhances apoptosis. Cell Growth Differ.

[b15] Lazarini F, Mouthon MA, Gheusi G, de Chaumont F, Olivo-Marin JC, Lamarque S, Abrous DN, Boussin FD, Lledo PM (2009). Cellular and behavioral effects of cranial irradiation of the subventricular zone in adult mice. PLoS ONE.

[b16] Louis SA, Rietze RL, Deleyrolle L, Wagey RE, Thomas TE, Eaves AC, Reynolds BA (2008). Enumeration of neural stem and progenitor cells in the neural colony forming cell assay. Stem Cells.

[b17] Lugert S, Basak O, Knuckles P, Haussler U, Fabel K, Gotz M, Haas CA, Kempermann G, Taylor V, Giachino C (2010). Quiescent and active hippocampal neural stem cells with distinct morphologies respond selectively to physiological and pathological stimuli and aging. Cell Stem Cell.

[b18] Maslov AY, Barone TA, Plunkett RJ, Pruitt SC (2004). Neural stem cell detection, characterization, and age-related changes in the subventricular zone of mice. J Neurosci.

[b19] Massague J (2012). TGFbeta signalling in context. Nat Rev Mol Cell Biol.

[b20] Mathieu C, Sii-Felice K, Fouchet P, Etienne O, Haton C, Mabondzo A, Boussin FD, Mouthon MA (2008). Endothelial cell-derived bone morphogenetic proteins control proliferation of neural stem/progenitor cells. Mol Cell Neurosci.

[b21] Mirzadeh Z, Merkle FT, Soriano-Navarro M, Garcia-Verdugo JM, Alvarez-Buylla A (2008). Neural stem cells confer unique pinwheel architecture to the ventricular surface in neurogenic regions of the adult brain. Cell Stem Cell.

[b22] Monje ML, Palmer T (2003). Radiation injury and neurogenesis. Curr Opin Neurol.

[b23] Monje ML, Toda H, Palmer TD (2003). Inflammatory blockade restores adult hippocampal neurogenesis. Science.

[b24] Morshead CM, Craig CG, van der Kooy D (1998). In vivo clonal analyses reveal the properties of endogenous neural stem cell proliferation in the adult mammalian forebrain. Development.

[b25] Mouthon M-A, Fouchet P, Mathieu C, Sii-Felice K, Etienne O, Silva Lages C, Boussin FD (2006). Neural stem cells from mouse forebrain are contained in a population distinct from the ‘side population’. J Neurochem.

[b26] Okabe M, Ikawa M, Kominami K, Nakanishi T, Nishimune Y (1997). ‘Green mice’ as a source of ubiquitous green cells. FEBS Lett.

[b27] Pastrana E, Cheng LC, Doetsch F (2009). Simultaneous prospective purification of adult subventricular zone neural stem cells and their progeny. Proc Natl Acad Sci USA.

[b28] Prediger RD, Aguiar AS, Rojas-Mayorquin AE, Figueiredo CP, Matheus FC, Ginestet L, Chevarin C, Bel ED, Mongeau R, Hamon M (2010). Single intranasal administration of 1-methyl-4-phenyl-1,2,3,6-tetrahydropyridine in C57BL/6 mice models early preclinical phase of Parkinson's disease. Neurotox Res.

[b29] Ramirez-Castillejo C, Sanchez-Sanchez F, Andreu-Agullo C, Ferron SR, Aroca-Aguilar JD, Sanchez P, Mira H, Escribano J, Farinas I (2006). Pigment epithelium-derived factor is a niche signal for neural stem cell renewal. Nat Neurosci.

[b30] Schober A, Peterziel H, von Bartheld CS, Simon H, Krieglstein K, Unsicker K (2007). GDNF applied to the MPTP-lesioned nigrostriatal system requires TGF-beta for its neuroprotective action. Neurobiol Dis.

[b31] Seoane J (2009). TGFbeta and cancer initiating cells. Cell Cycle.

[b32] Seoane J, Le HV, Shen FL, Anderson SA, Massague J (2004). Integration of Smad and forkhead pathways in the control of neuroepithelial and glioblastoma cell proliferation. Cell.

[b33] Shen Q, Wang Y, Kokovay E, Lin G, Chuang SM, Goderie SK, Roysam B, Temple S (2008). Adult SVZ stem cells lie in a vascular niche: a quantitative analysis of niche cell-cell interactions. Cell Stem Cell.

[b34] Sii-Felice K, Etienne O, Hoffschir F, Mathieu C, Riou L, Barroca V, Haton C, Arwert F, Fouchet P, Boussin FD (2008). Fanconi DNA repair pathway is required for survival and long-term maintenance of neural progenitors. EMBO J.

[b35] Sirerol-Piquer MS, Cebrian-Silla A, Alfaro-Cervello C, Gomez-Pinedo U, Soriano-Navarro M, Garcia-Verdugo JM (2012). GFP immunogold staining, from light to electron microscopy, in mammalian cells. Micron.

[b36] Tada E, Yang C, Gobbel GT, Lamborn KR, Fike JR (1999). Long-term impairment of subependymal repopulation following damage by ionizing irradiation. Exp Neurol.

[b37] Tavazoie M, Van der Veken L, Silva-Vargas V, Louissaint M, Colonna L, Zaidi B, Garcia-Verdugo JM, Doetsch F (2008). A specialized vascular niche for adult neural stem cells. Cell Stem Cell.

[b38] Trnovec T, Kallay Z, Bezek S (1990). Effects of ionizing radiation on the blood brain barrier permeability to pharmacologically active substances. Int J Radiat Oncol Biol Phys.

[b39] Tropepe V, Craig CG, Morshead CM, van der Kooy D (1997). Transforming growth factor-alpha null and senescent mice show decreased neural progenitor cell proliferation in the forebrain subependyma. J Neurosci.

[b40] Valley MT, Mullen TR, Schultz LC, Sagdullaev BT, Firestein S (2009). Ablation of mouse adult neurogenesis alters olfactory bulb structure and olfactory fear conditioning. Front Neurosci.

[b41] Villeda SA, Luo J, Mosher KI, Zou B, Britschgi M, Bieri G, Stan TM, Fainberg N, Ding Z, Eggel A (2011). The ageing systemic milieu negatively regulates neurogenesis and cognitive function. Nature.

[b42] Wachs FP, Winner B, Couillard-Despres S, Schiller T, Aigner R, Winkler J, Bogdahn U, Aigner L (2006). Transforming growth factor-beta1 is a negative modulator of adult neurogenesis. J Neuropathol Exp Neurol.

[b43] Werry EL, Enjeti S, Halliday GM, Sachdev PS, Double KL (2010). Effect of age on proliferation-regulating factors in human adult neurogenic regions. J Neurochem.

[b44] Winkler EA, Bell RD, Zlokovic BV (2011). Central nervous system pericytes in health and disease. Nat Neurosci.

[b45] Wyss-Coray T, Lin C, Sanan DA, Mucke L, Masliah E (2000). Chronic overproduction of transforming growth factor-beta1 by astrocytes promotes Alzheimer's disease-like microvascular degeneration in transgenic mice. Am J Pathol.

[b46] Yingling JM, Blanchard KL, Sawyer JS (2004). Development of TGF-beta signalling inhibitors for cancer therapy. Nat Rev Drug Discov.

