# Supplementary figures and images for: Vascular-derived TGF-β increases in the stem cell niche and perturbs neurogenesis during aging and following irradiation in the adult mouse brain
Source: EMBO Mol Med. 2013 Mar 25;5(4):548–62. doi: 10.1002/emmm.201202197 (PMC3628106; doi:10.1002/emmm.201202197)

# Source data Figure 5

C

Running buffer Tris-Glycine

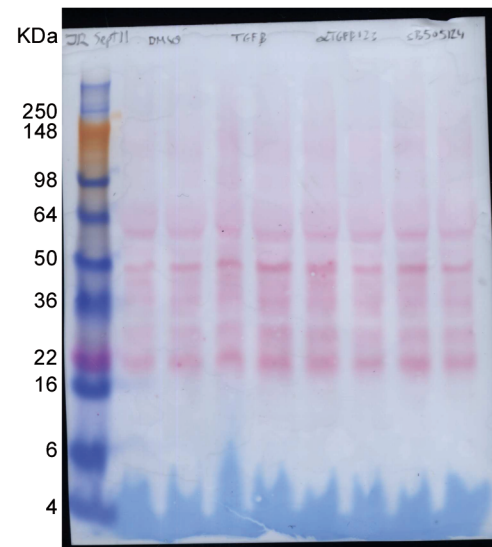

Ponceau

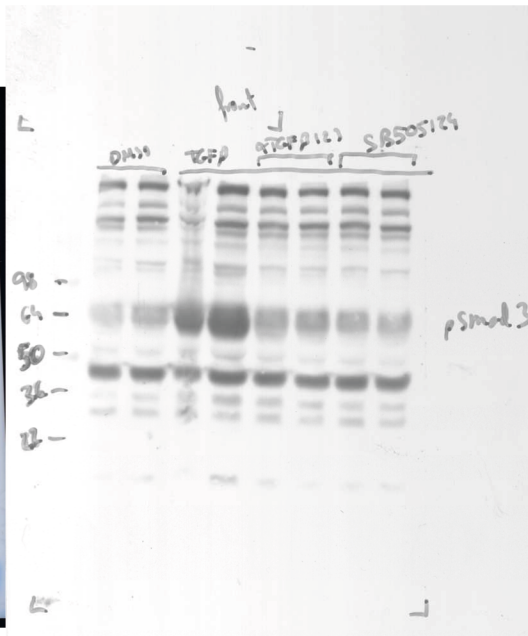

P-Smad3

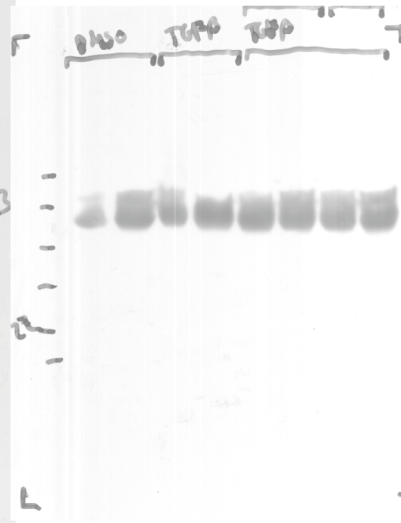

Total Smad 2/3

Supplement: Supplementary file 2 [file emmm0005-0548-sd2.pdf]

# Source data Figure 6

A

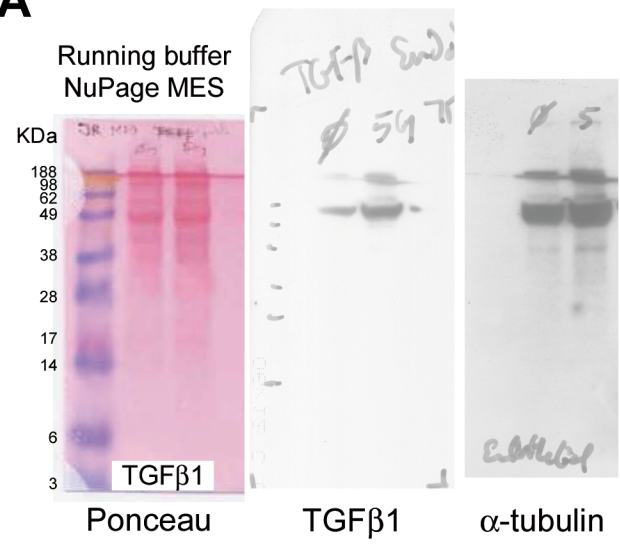

B

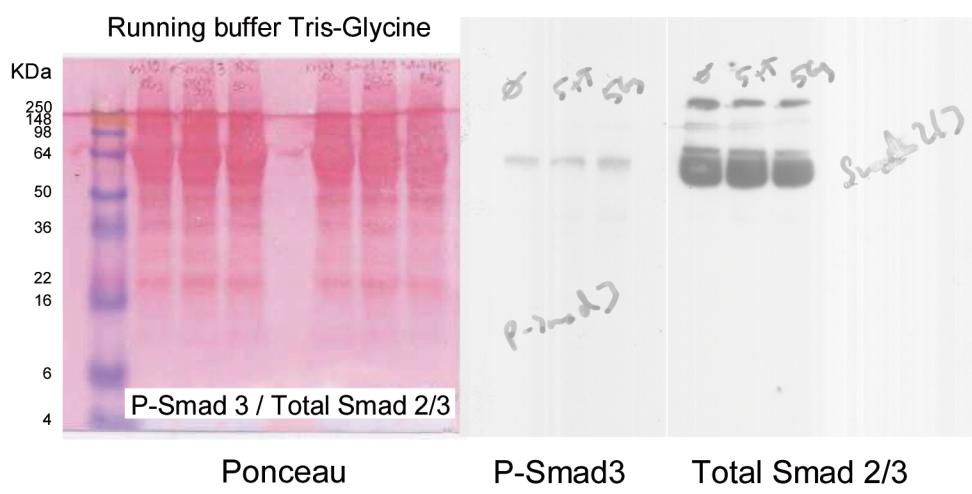

Supplement: Supplementary file 3 [file emmm0005-0548-sd3.pdf]
