# Supplementary material for: Vascular-derived TGF-β increases in the stem cell niche and perturbs neurogenesis during aging and following irradiation in the adult mouse brain
Source: EMBO Mol Med. 2013 Mar 25;5(4):548–62. doi: 10.1002/emmm.201202197 (PMC3628106; doi:10.1002/emmm.201202197)
Supplement: Supplementary file 4 [file emmm0005-0548-sd4.pdf]

Supplementary informations for:

Vascular-derived TGF-β increases in the stem cell niche and perturbs neurogenesis during aging and following irradiation in the adult mouse brain

Jose R. PINEDA, Mathieu DAYNAC, Alexandra CHICHEPORTICHE, Arantxa CEBRIAN-SILLA, Karine SII FELICE, Jose Manuel GARCIA-VERDUGO, François D. BOUSSIN and Marc-André MOUTHON

**Supplementary information**	**Content**
Supplementary Figure1:	Split dose radiation does not mobilize microglial cells
Supplementary Figure 2:	Cells that resemble NSCs are present in the irradiated brain
Supplementary Figure 3:	Irradiation blocks proliferation in the SVZ
Supplementary Figure 4:	FACS strategy for NSC sorting and analysis
Supplementary Figure 5:	NSCs with a GLAST+CD24- phenotype are maintained in the SVZ following irradiation
Supplementary Figure 6:	Grafted NSCs differentiate into neurons and migrate in the OBs
Supplementary Figure 7:	Phospho-Smad2 is undetectable in the SVZ
Supplementary Figure 8:	Irradiated BECs induce neural progenitor apoptosis through TGFβ
Supplementary Figure 9:	The inhibitory effect of TGFβ1 on neurosphere growth is blocked by treatment with an anti-TGFβ blocking antibody
Supplementary Figure 10:	Alteration of mural coverage with anti-TGFβ therapy
Supplementary Figure 11:	SB-505124 increases production of neuroblasts and proliferation of NSCs in both irradiated and elderly mice
Supplementary Table 1:	Phenotype of TGFβ-positive cells in SVZ
Supplementary Table 2:	P-values for TGFβ-binding on SVZ cells as compared to young adult mice
Supplementary Table 3:	Primary antibodies
Supplementary Table 4:	Primer sequences

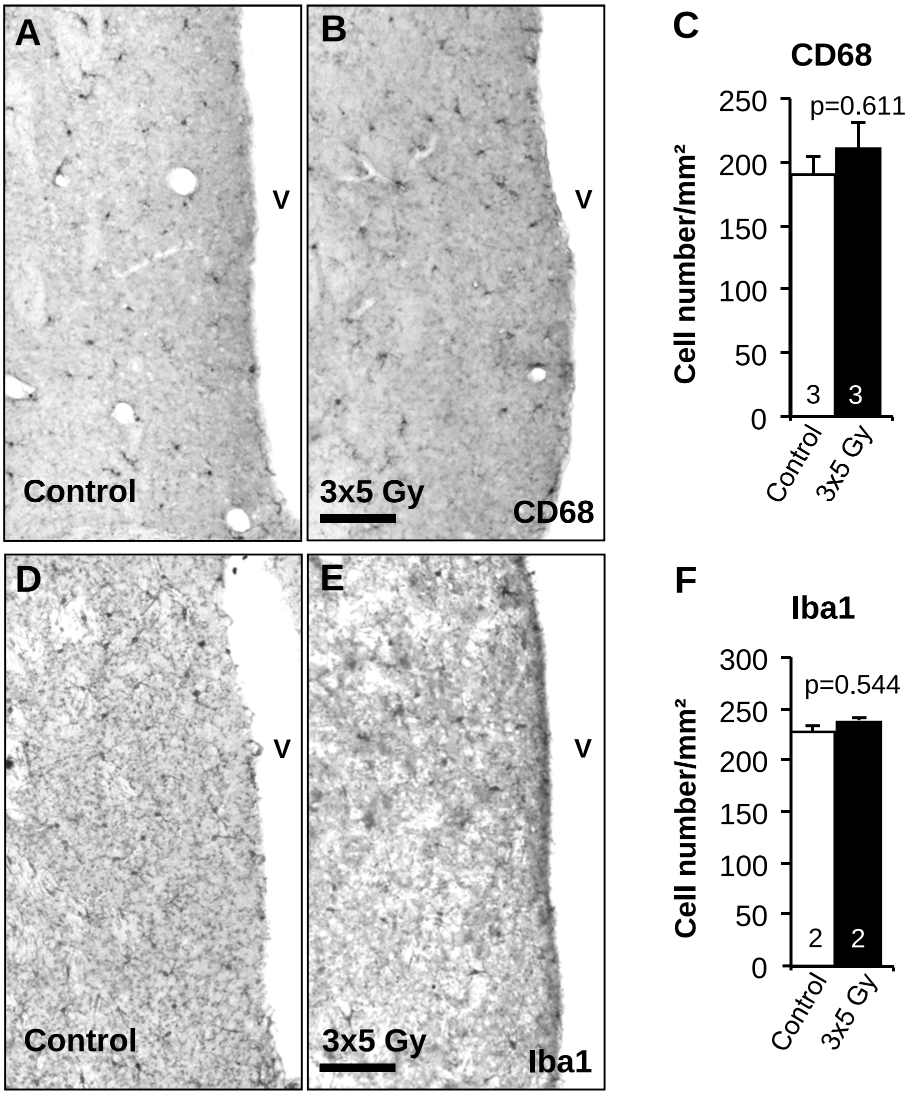

Supplementary Figure 1: Split dose radiation does not mobilize microglial cells

Immunohistochemistry for CD68 (A-C) and Iba1 (D-F) revealed microglial cells in a region that encompassed the proximal striatum and the SVZ of control (A and D) and irradiated mice 2 months following exposure (B and E). Quantifications (mean ± s.d.) of CD68-positive cells (C) and Iba1 (F) revealed that the 3x5 Gy split-exposure does not mobilise microglial cells into the brain. Scale bars = 100µm. V: ventricle. The *p*-value was determined using the Mann–Whitney U-test. The number of mice is indicated within bars.

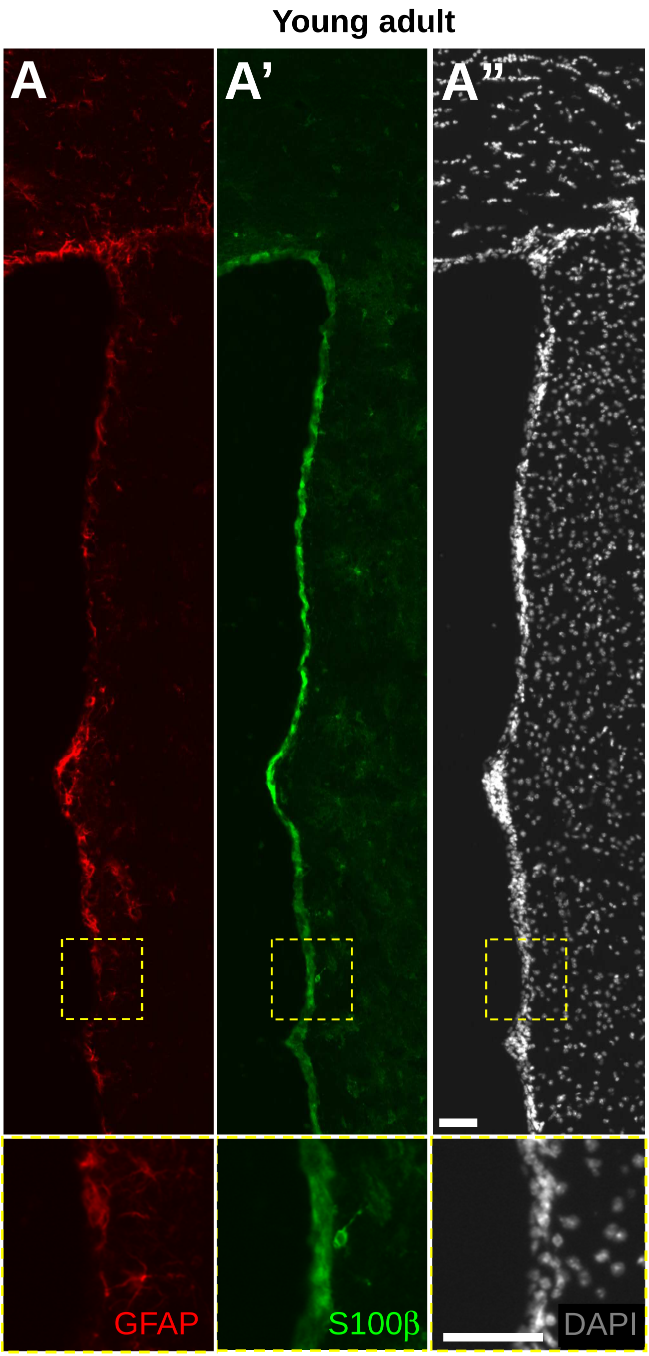

Supplementary Figure 2: Cells that resemble NSCs are present in the irradiated brain

In control mice, NSCs lining lateral ventricle are characterised by their positivity for GFAP (A) and negativity for S100β (A') a marker associated with differentiated astrocytes and ependymal cells. Certain NSCs survived in the SVZ 4 months following irradiation (B-B"). The survival of astrocytic-like NSCs was confirmed by nestin/GFAP double immunostaining (C). Scale bars = 100μm.

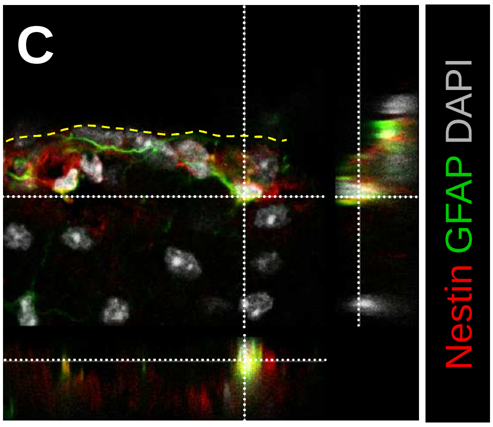

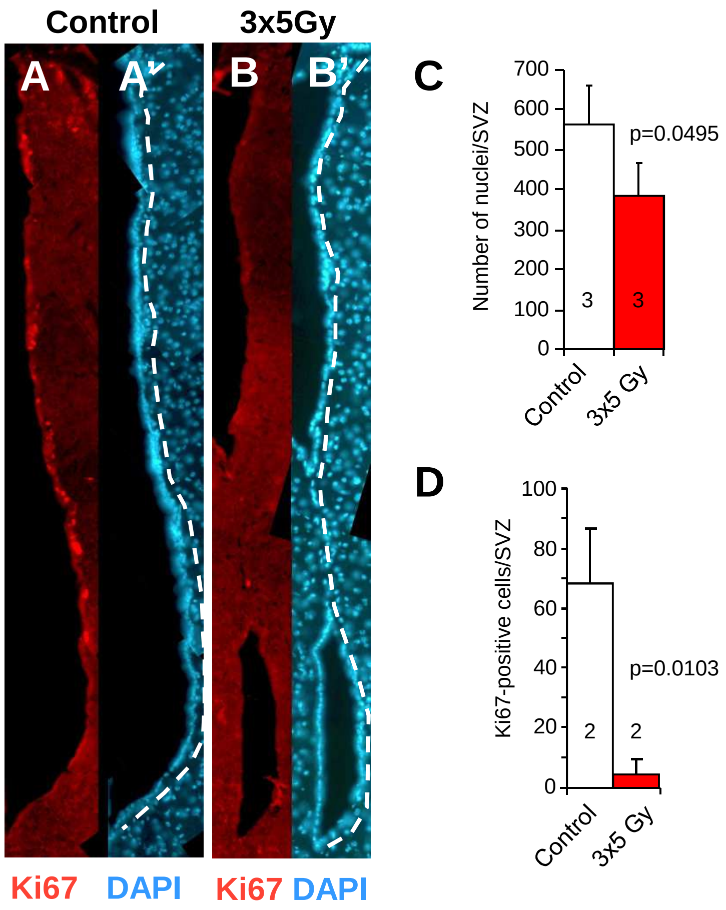

Supplementary Figure 3: Irradiation blocks proliferation in the SVZ

Ki-67 immunostaining and nuclei staining with DAPI indicated that cell proliferation was profoundly reduced in the SVZ 4 months following exposure (B, B') in comparison to the control mice (A, A'). The mean ± s.d. are shown in C and D. The p-value was determined using the Mann–Whitney U-test. The number of mice is indicated within bars.

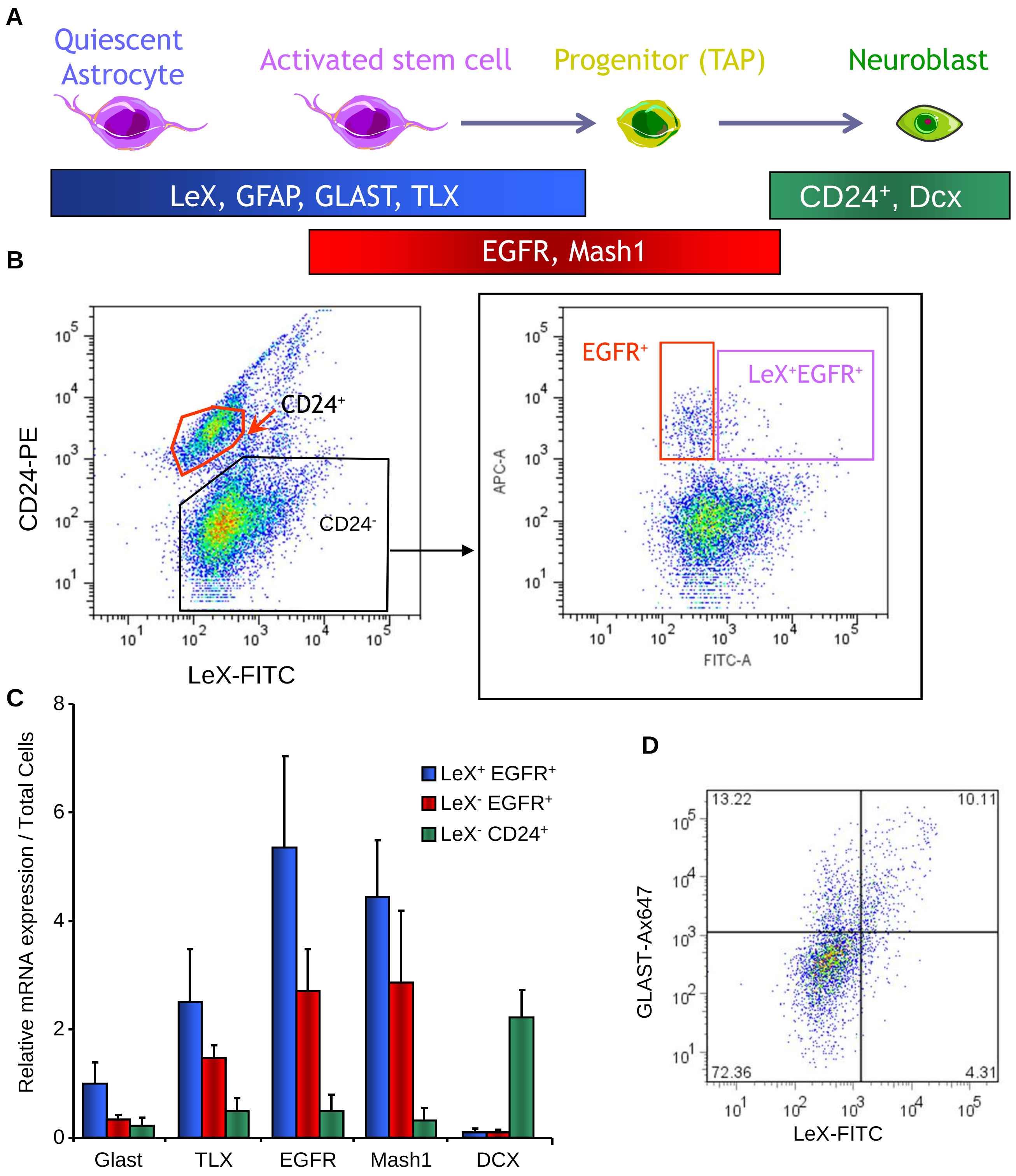

Supplementary Figure 4: FACS strategy for NSC sorting and analysis

(A) Schematic representation of NSCs and their progeny in the SVZ, and the expression of several markers. (B) Sorting gates are represented for the neuroblasts ($CD24^+$), activated NSCs ($CD24^-$ LeX^+EGFR^+) and TAPs ($CD24^-LeX^+EGFR^-$). (C) The expression of specific markers for NSCs, TAPs and neuroblasts was examined for sorted populations by qRT-PCR. The mean ± s.d. were obtained from pooled samples with n = 8-10 mice in three independent sorting experiments. (D) FACS analysis of $CD24^-$ cells in the SVZ showed that nearly all of the LeX+ cells express GLAST, although a subset of $GLAST^+$ cells were observed to be LeX-negative.

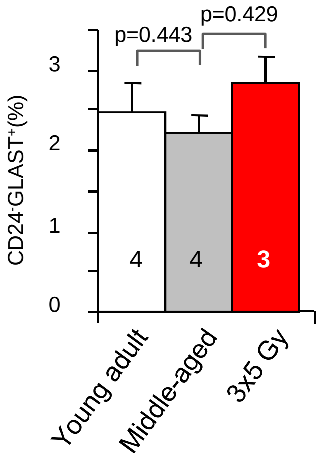

Supplementary Figure 5: NSCs with a $CD24^{-}GLAST^{+}$ phenotype are maintained in the SVZ following irradiation and during aging

The quantification of $CD24^{-}GLAST^{+}$ in the SVZ by FACS in young adult (2-4 months), middle-aged (12 months) and young adult mice 3-4 months after the 3x5 Gy split-dose irradiation. The mean ± s.d. is shown (the number of mice is indicated within the bars). The *p*-value was determined using the Mann–Whitney U-test.

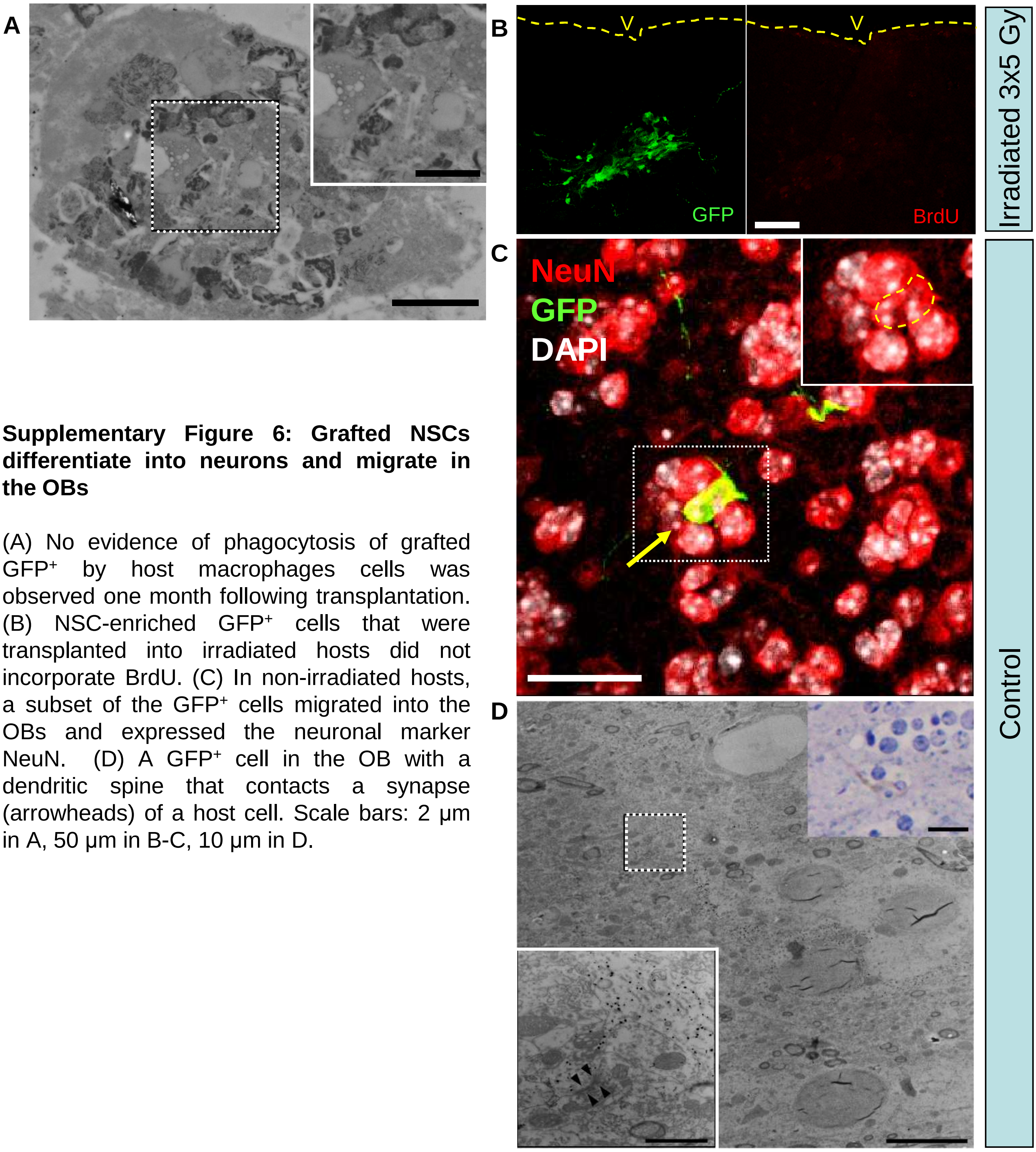

Supplementary Figure 6: Grafted NSCs differentiate into neurons and migrate in the OBs

(A) No evidence of phagocytosis of grafted GFP^{+} by host macrophages cells was observed one month following transplantation. (B) NSC-enriched GFP^{+} cells that were transplanted into irradiated hosts did not incorporate BrdU. (C) In non-irradiated hosts, a subset of the GFP^{+} cells migrated into the OBs and expressed the neuronal marker NeuN. (D) A GFP^{+} cell in the OB with a dendritic spine that contacts a synapse (arrowheads) of a host cell. Scale bars: 2 μm in A, 50 μm in B-C, 10 μm in D.

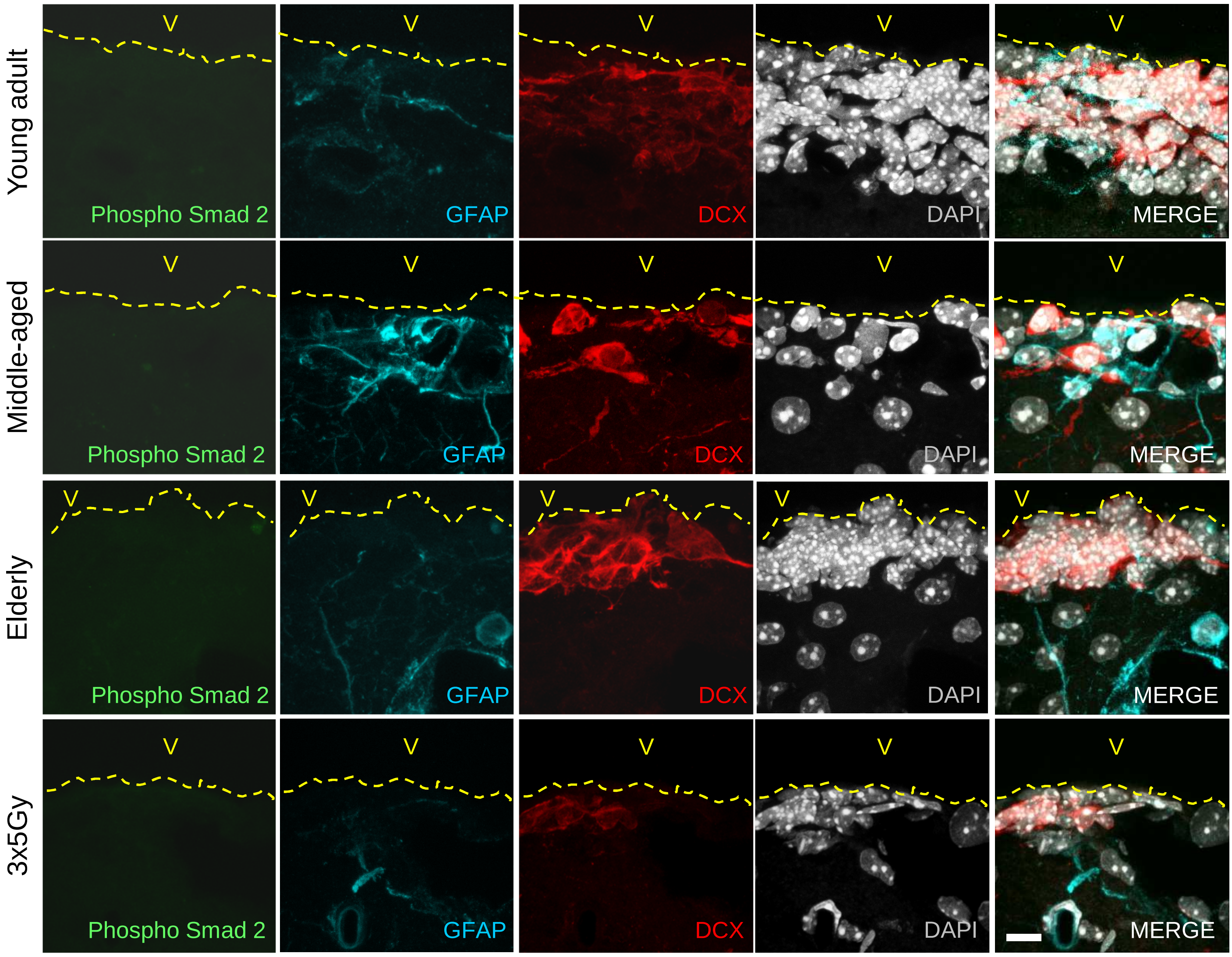

Supplementary Figure 7: Phospho-Smad2 is undetectable in the SVZ

Triple immunostaining for phospho-Smad 2, GFAP and doublecortin (DCX) allows for the examination of neurogenic niches in the SVZ (V: lateral ventricle). Smad2 phosphorylation was undetectable. The illustrations are representative of three different experiments, with three mice per group.
Scale bar = 10µm.

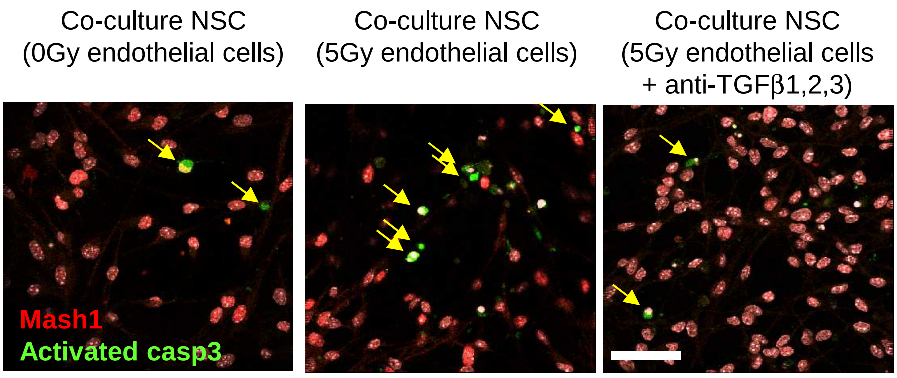

Supplementary Figure 8: Irradiated BECs induce neural progenitor apoptosis via TGFβ

Apoptosis (activated caspase 3) of Mash1-positive neural stem/progenitor cells in co-culture with irradiated endothelial cells was reduced following treatment with a blocking anti-TGFβ antibody. Scale bar = 50 μm.

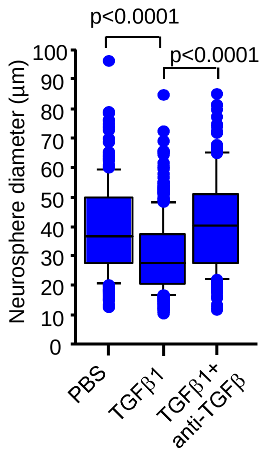

Supplementary Figure 9: The inhibitory effect of TGFβ1 on neurosphere growth is blocked by treatment with an anti-TGFβ blocking antibody

The neurosphere size was determined 7 days following the addition of TGFβ1 (1 ng/ml) in the presence or absence of an anti-TGFβ blocking antibody. The mean ± s.d. was obtained from at least 248 neurospheres per condition in two individual cultures. The *p*-value was determined using Student's *t*-test.

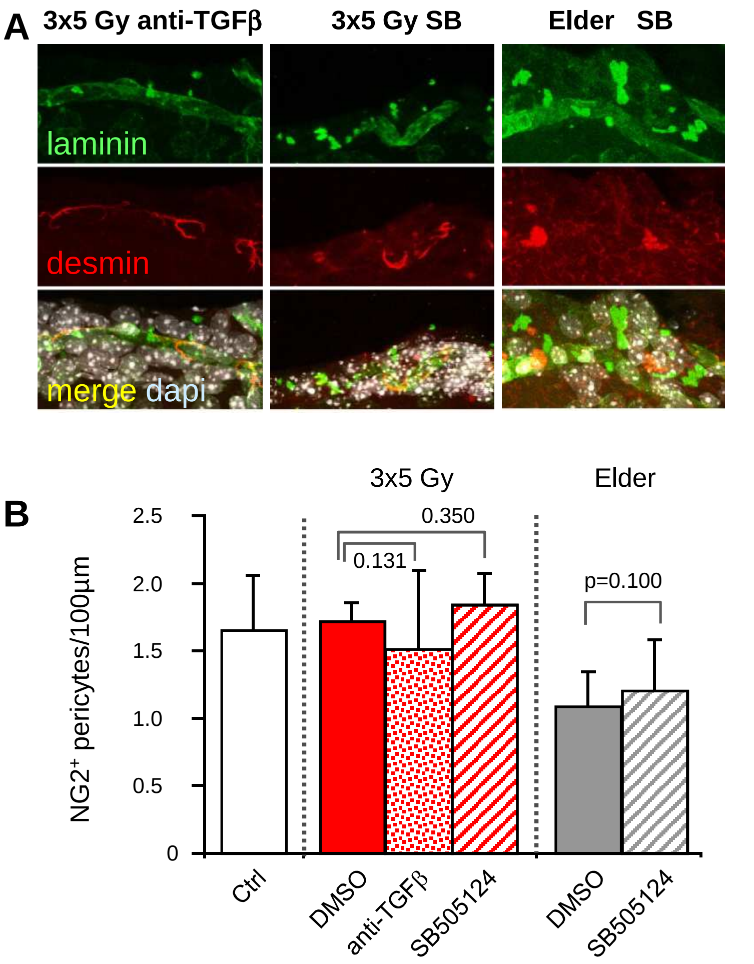

Supplementary Figure 10: Alteration of mural coverage with anti-TGFβ therapy

(A) The effects SB-505124 or of the anti-TGFβ blocking antibody were examined on mural coverage of SVZ capillaries using desmin/laminin immunostainings. (B) NG2/pericytes were quantified on SVZ capillaries. Scale bar: 50 µm. The mean ± s.d. of two independent experiments is shown. The *p*-value was determined using the Mann–Whitney U-test.

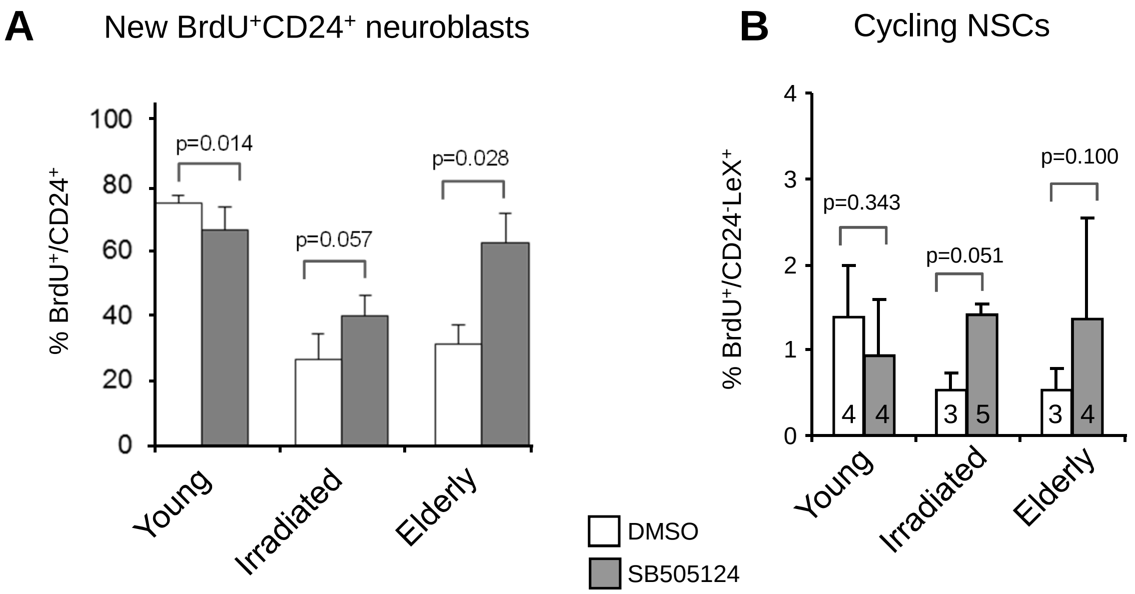

Supplementary Figure 11: SB-505124 increases production of neuroblasts and proliferation of NSCs in both irradiated and elderly mice

SB-505124 was administered for 5 days in young adult, irradiated or elderly mice. The mice were euthanised one day after the final treatment. The incorporation of BrdU was determined by FACS analysis of $CD24^+$ neuroblasts (A) and of $CD24^-LeX^+$ NSCs (B). The mean ± s.d. of two independent experiments is shown (the number of mice is indicated within bars). The *p*-value was determined using the Mann–Whitney U-test.

Supplementary Table 1: Phenotype of TGFβ-positive cells in SVZ

Cycling DNA>2N	Nblast $CD24^+$	TAPs EGF^+	Activated NSCs $LeX^+EGF^+CD24^-$	NSCs $GLAST^+CD24^-$
4 ± 1%	3 ± 2%	17 ± 2%	48 ± 2%	27 ± 4%

Supplementary Table 2: P-values for TGFβ-binding on SVZ cells compared to young adult mice

	**Middle-aged**	**Elder**	**3x5 Gy**
**Neuroblats**	0.564	0.564	>0.999
**Cycling**	0.147	0.020	0.020
**TAPs**	>0.999	0.439	0.439
**NSCs**	0.121	0.121	0.439

Supplementary Table 3: Primary antibodies

Target	Host	Clone/ref	Dilution	Provider
**GFAP**	Ms	GA5	400	**Millipore**
**Id1**	Ms	7D4	100	**Millipore**
**NeuN**	Ms	A60	100	**Millipore**
**Sox2**	Ms	6F1.2	100	**Millipore**
**Cyclin D1**	Rb	06-137	200	**Millipore**
**p21waf**	Ms	Clone 65	100	**Millipore**
**Smad2/3**	Rb	071408	2000[a]	**Millipore**
**GFP**	Rb	Ab290	200/1000[b]	**Abcam**
**TGFb1**	Ms	2Ar2	50/500[a]	**Abcam**
**phospho-Smad3 (S423/425)**	Rb	ab51451	100/1000[a]	**Abcam**
**TGFb receptor I**	Rb	ab31013	100	**Abcam**
**TGFb receptor II**	Rb	ab61213	100	**Abcam**
**phospho-Smad2 (S465/467)**	Rb	mAb3101	100	**Cell Signalling**
**cleaved caspase-3**	Rb	mAb9579	100	**Cell Signalling**
**Laminin**	Rb	L9393	50	**Sigma-Aldrich**
**Desmin**	Ms	D33	150	**Dako**
**NG2**	Ms	AB5320	150	**Millipore**
**a-tubulin**	Ms	DM1A	1000[a]	**Sigma-Aldrich**
**GFAP**	Rb	G9269	400	**Sigma-Aldrich**
**bIII-tubulin**	Rb	PRB-435P	200	**Covance**
**Ki-67**	Ms	MM1	100	**Covance**
**Nestin**	Ms	Rat401	200	**Becton Dickinson**
**Mash1**	Ms	24B7.2D11	50	**Becton Dickinson**
**CD15/LeX**	Ms	MMA	100	**Becton Dickinson**
**CD68**	Rat	FA-11	100	**AbDSerotec**
**S100b**	Rb	Z0311	200	**Dako**
**BrdU**	Ms	RPN202	300	**GE Healthcare**
**Doublecortin**	Goat	C-18	200	**SantaCruz**
**CD24-PE**	Rat	30-F1	500	**Becton Dickinson**
**LeX/CD15-FITC**	Ms	MMA	50	**Becton Dickinson**
**CD31-PE**	Rat	MEC13.3	50	**Becton Dickinson**
**CD45-PC5 or PE**	Rat	30-F11	50	**Becton Dickinson**
**GLAST**	Rat	ACSA-1	50	**Miltenyi**

[a]Dilution is given for western blot. [b]Dilution is given for DAB revelation.

Supplemental Table 4: Primer sequences

Target gene	Forward primer 5’ → 3’	Reverse primer 5’ → 3’
**Gapdh**	CCAGTATGACTCCACTCACG	GACTCCACGACATACTCAGC
**18S**	ATACATGCCGACGGGCGCTG	AGGGAGCTCACCGGGTTGGTT
**Tlx**	ATGCCCCGTAGACAAGACAC	CGGAAGTAGAGAGCCACCTG
**Mash1**	TGTCTTTCCTCAGTCACCCC	GAAAGGCTGTCCGAGAACTG
**Dcx**	TCCCCAACACCTCAAAAGAC	TTGAGAGCTGACTGCTGGAA
**TGFβ1**	GGTGTCAGAGCCTCACCGCG	AGAGCGGGAACCCTCGGCAA

Primers were purchased from Eurogentec.
